# Application of Dual-Targeting Carbon Dot Nanozymes in Improving Hypoxia and Enhancing Small Interfering RNA-Mediated Gene Silencing for Hepatocellular Carcinoma

**DOI:** 10.34133/bmr.0430

**Published:** 2026-10-19

**Authors:** Yingying Wei, Yangyi Cao, Wencheng Shao, Tao Zhang, Xiaobo Zhang, Wenhui Dong, Shizhao Zhou, Yongzhen Yang, Lin Chen, Shiping Yu

**Affiliations:** ^1^ Department of Interventional Therapy, Shanxi Province Cancer Hospital/Shanxi Hospital Affiliated to Cancer Hospital, Chinese Academy of Medical Sciences/ Cancer Hospital Affiliated to Shanxi Medical University, Taiyuan 030000, P. R. China.; ^2^ Affiliated Cancer Hospital, Shanxi Medical University, Taiyuan 030000, P. R. China.; ^3^ The Department of Radiology, Renmin Hospital of Wuhan University, Wuhan 430060, P. R. China.; ^4^ Medical Imaging Department, Shanxi Medical University, Taiyuan 030001, P. R. China.; ^5^ Shanxi Academy of Medical Sciences, Shanxi Medical University, Taiyuan 030001, P. R. China.; ^6^ Department of Radiology, The First Medical Center of PLA General Hospital, Beijing 100039, P. R. China.; ^7^ Key Laboratory of Interface Science and Engineering in Advanced Materials, Ministry of Education, Taiyuan University of Technology, Taiyuan 030024, P. R. China.; ^8^ College of Textile Engineering, Taiyuan University of Technology, Taiyuan 030024, P. R. China.

## Abstract

Hepatocellular carcinoma (HCC) is a malignant tumor with high morbidity and mortality, and current therapeutic effects remain unsatisfactory. Small interfering RNA (siRNA) therapy has attracted widespread attention owing to its strong specificity and high silencing efficiency. Golgi apparatus (GA) protein 73 (GP73) is overexpressed in HCC and promotes proliferation and invasion of HCC. However, the hypoxic microenvironment of HCC facilitates GP73 synthesis. Based on the bidirectional regulation between GP73 and the hypoxic microenvironment, this study proposes the use of carbon dot nanozyme with dual-targeting properties to deliver GP73-siRNA to the GA of HCC cells, thereby inhibiting GP73 expression and simultaneously alleviating hypoxia for synergistic HCC treatment and improved efficacy. Specifically, GA targeting manganese-doped carbon dot nanozyme (Mn-CDs) was constructed using neutral red (with a large sp^2^ conjugated structure), L-cysteine (GA targeting agent), and manganese dioxide as raw materials via a one-step solvothermal method. Polyethyleneimine (PEI) and hyaluronic acid (HA) were modified on the surface of Mn-CDs to obtain the dual-targeting Mn-CDs-PEI-HA. Subsequently, Mn-CDs-PEI-HA was used as a carrier to load GP73-siRNA and form the Mn-CDs-PEI-HA/GP73-siRNA complex. Results demonstrated that the complex exhibited excellent catalase-like enzyme activity, which can alleviate tumor hypoxia, and could deliver GP73-siRNA to the GA of HCC cells, thereby silencing GP73 expression and synergistically inhibiting HCC progression. The therapeutic effect under hypoxic conditions was superior to that under normoxic conditions. Additionally, by simultaneously taking advantage of the favorable fluorescence and magnetic properties of Mn-CDs-PEI-HA, fluorescence/magnetic resonance dual-modality imaging of HCC was achieved, providing a new strategy for theranostics of HCC.

## Introduction

Hepatocellular carcinoma (HCC) is a type of malignant tumor with high global incidence and mortality rates worldwide, posing a serious threat to human life and health [1]. Although current treatment methods are continuously improving, there is still the challenge of HCC constantly proliferating, recurring, and metastasizing. Therefore, exploring novel therapeutic approaches to enhance treatment efficacy is crucial. With the advancement of genome editing technologies, small interfering RNA (siRNA) therapy has shown great promise in treating diseases such as cancer, owing to its strong specificity, minimal side effects, high silencing efficiency, and easily detectable therapeutic outcomes [2]. siRNA is a nucleic-acid-based drug that, upon delivery into target cells, binds to messenger RNA (mRNA) in a sequence-specific manner and cleaves it, thereby down-regulating mRNA expression and reducing the synthesis of specific proteins [3]. The Golgi apparatus (GA), as the primary site responsible for protein modification, sorting, packaging, and transport, has its surface protein abnormalities closely associated with tumor development and progression [4]. Therefore, using siRNA to target and inhibit protein synthesis on the GA is beneficial for improving HCC treatment efficacy. However, naked siRNA has a short in vivo half-life, is susceptible to degradation by RNase and systemic clearance, struggles to cross vascular barriers and accumulate in tumor tissues, and faces even greater challenges in cellular uptake and subsequent endosomal escape. As a result, the efficiency of siRNA entering the cytoplasm, combining with RNA-induced silencing complex and exerting RNA interference (RNAi), is extremely low. Thus, constructing an efficient delivery system is crucial for protecting siRNA from RNase degradation and systemic clearance, promoting its accumulation in tumor tissues, enhancing its cytoplasmic delivery capacity after cellular uptake, achieving effective endosomal escape, and ultimately enabling RNAi and tumor therapy.

GA protein 73 (GP73), a transmembrane glycoprotein (molecular weight 73×10^3^ Da) located in the GA, is primarily expressed in epithelial cells of various human tissues, but shows minimal or no expression in normal liver tissue, while being overexpressed in HCC tissues [5]. Studies have demonstrated that GP73 overexpression alters glycosphingolipid synthesis and plasma membrane composition, thereby promoting mitotic activity and cell proliferation in HCC cells [6]. Concurrently, under the hypoxic microenvironment of HCC, GP73 synthesis is enhanced, further facilitating HCC invasion and metastasis [7]. In summary, both hypoxia and GP73 play critical roles in HCC progression. Consequently, on the basis of ameliorating hypoxia, the application of GP73-siRNA combined with an efficient delivery vector is expected to overcome the hypoxic microenvironment while precisely knocking down GP73 expression, thereby breaking through the current therapeutic bottlenecks and providing a novel and synergistically enhanced RNAi strategy for HCC.

At present, a variety of nonviral nanocarriers for siRNA delivery have been constructed, including carbon nanoparticles, lipid nanoparticles, metal nanoparticles, cell-membrane-coated nanoparticles, and cationic polymeric nanoparticles, among others. Compared with other nanocarriers, carbon dots (CDs), a type of zero-dimensional carbon nanoparticle with a size less than 10 nm, are widely used in gene therapy, photodynamic therapy, photothermal therapy, sonodynamic therapy, and biological imaging for tumors [8–11] due to their advantages such as low toxicity, good biocompatibility, abundant surface functional groups, ease of modification, and excellent fluorescence (FL) properties [12]. CDs carrying siRNA can effectively silence mRNA expression, reduce the synthesis of corresponding proteins, and inhibit tumor growth while enabling biofluorescence imaging [13]. Xue et al. [14] used CDs modified with polyethyleneimine (PEI), which possesses high transfection efficiency, as siRNA carriers to deliver survivin siRNA, improving the transfection efficiency of siRNA, achieving efficient siRNA delivery, and enhancing the therapeutic effect on HCC. Furthermore, doping CDs with manganese (Mn) endowed them with catalase (CAT) activity, enabling them to catalyze hydrogen peroxide to produce oxygen (O_2_) and alleviate tumor hypoxia. Simultaneously, CDs are endowed with magnetism and can be applied to magnetic resonance (MR) imaging. Therefore, CD-based nanozymes can serve as siRNA carriers to deliver GP73-siRNA, simultaneously ameliorating hypoxia and down-regulating GP73 expression, thereby achieving dual-modality imaging and dual therapy for HCC.

In this study, Mn-CDs were synthesized by a one-step solvothermal method using neutral red (NR), L-cysteine (L-Cys; GA targeting agent [15]), and MnO_2_ (Mn source) as raw materials. Mn-CDs exhibit excellent FL properties, magnetism, CAT-like activity, and GA targeting capability. Subsequently, PEI-hyaluronic acid (HA) was modified on the surface of Mn-CDs to enable active targeting of HCC cells, yielding dual-targeting Mn-CDs-PEI-HA, which was applied for FL/MR dual-modality imaging of HCC. Finally, the complex Mn-CDs-PEI-HA/GP73-siRNA was constructed using Mn-CDs-PEI-HA as a carrier to load GP73-siRNA. Mn-CDs-PEI-HA/GP73-siRNA enhances the therapeutic efficacy of HCC by simultaneously alleviating tumor hypoxia and down-regulating GP73 expression (Fig. 1), providing a novel strategy for combination therapy of HCC.

**Fig. 1.**
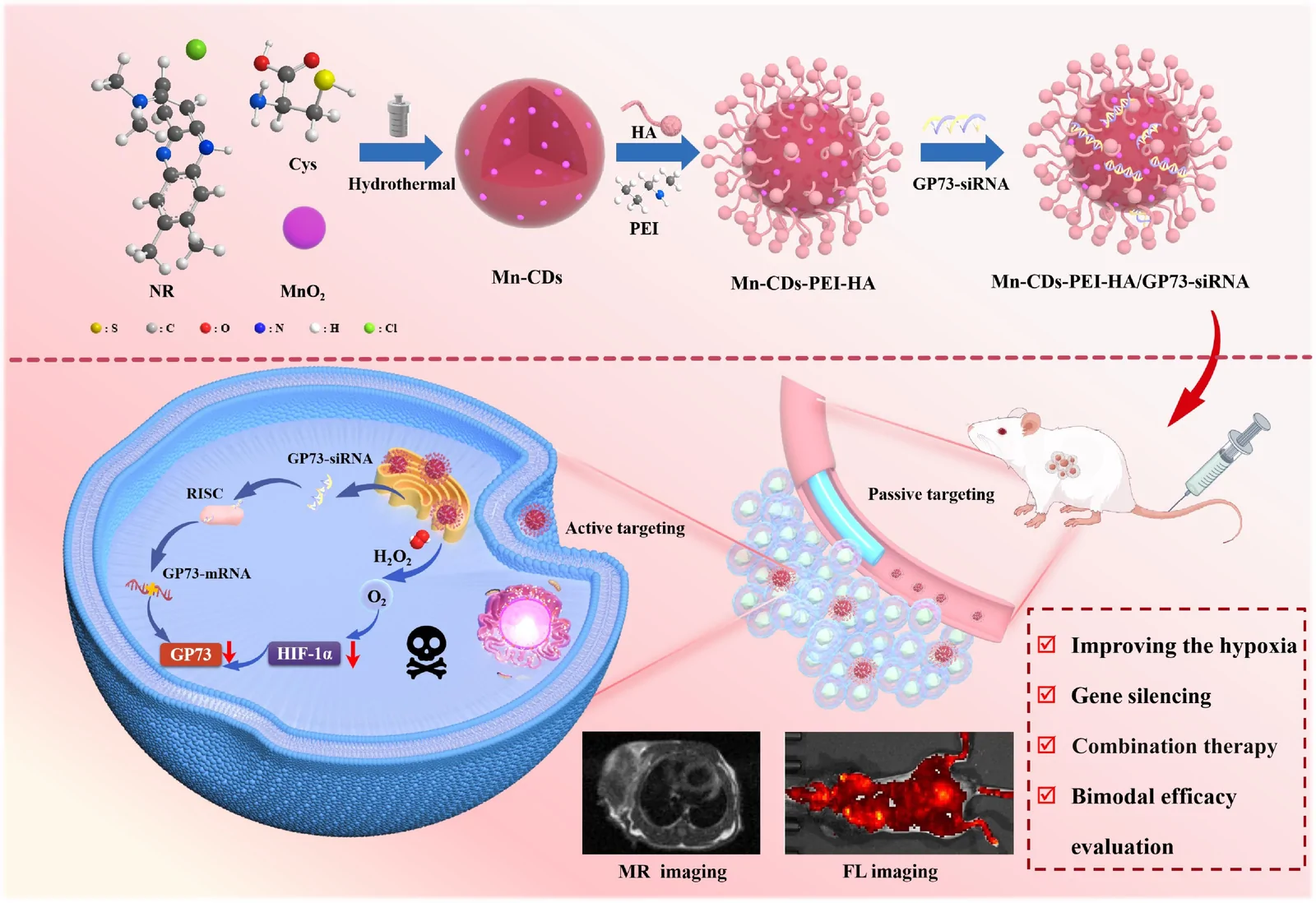
Schematic illustration of the preparation process of Mn-CDs-PEI-HA/GP73-siRNA and their application for dual-modality imaging and hepatocellular carcinoma (HCC) therapy. Cys, cysteine; FL, fluorescence; HA, hyaluronic acid; NR, nuclear red; MR, magnetic resonance; PEI, polyethyleneimine.

## Materials and Methods

### Materials

L-Cys, HA, PEI, and *tris* (4,7-diphenyl-1,10-phenanthroline) ruthenium (II) dichloride (Ru(dpp)_3_Cl_2_, RDPP) were purchased from Shanghai Macklin Biochemical Technology Co., Ltd. NR was purchased from Tianjin Dongli District Tianda Chemical Reagent Factory. Absolute ethanol was obtained from Sinopharm Chemical Reagent Co., Ltd. MnO_2_ and the amide activation agents 1-ethyl-(3-dimethylaminopropyl) carbodiimide hydrochloride (EDC) and *N*-hydroxysuccinimide (NHS) were procured from Shanghai Aladdin Biochemical Technology Co., Ltd. Cellulose dialysis bags (1,000 and 10,000 Da) were purchased from Shanghai Yuanye Bio-Technology Co., Ltd. Phosphate-buffered saline (PBS), Hank’s buffer, high-glucose basal medium (Dulbecco’s Modified Eagle Medium, DMEM), RPMI-1640 basal medium, human normal liver cells (THLE-2), human hepatocellular carcinoma cells (Huh-7), and mouse HCC cells (Hepa1-6) were all obtained from Wuhan Procell Life Science & Technology Co., Ltd. Trypsin, penicillin–streptomycin mixture, and crystal violet were sourced from Beijing Solarbio Science & Technology Co., Ltd. Fetal bovine serum (FBS) was purchased from Wuhan Servicebio Technology Co., Ltd. BALB/c nude mice and C57BL/6 mice (C57 mice) were acquired from Beijing Vital River Laboratory Animal Technology Co., Ltd. (Beijing, China). Tris–acetate–EDTA buffer, agarose, 6× DNA loading buffer, GelRed (10,000×) nucleic acid dye, live/dead cell staining kit, Matrigel, and cell counting kit-8 (CCK-8) reagent were all procured from Beijing Lab Lead Co., Ltd. GP73-siRNA was purchased from Shanghai Sangon Biotech Co., Ltd. Commercial GA green fluorescent dye (*N*-(NBD-aminohexanoyl) D-erythro-sphingosine, NBD) was obtained from Shanghai Maokang Biotechnology Co., Ltd. Nuclease-free diethyl pyrocarbonate (DEPC) water, paraformaldehyde fixative solution, and Transwell invasion chambers were purchased from Beijing Lanjie Ke Technology Co., Ltd. Hypoxia gas packs (1% O_2_ and 5% CO_2_) and hypoxia chambers were acquired from Mitsubishi Gas Chemical Company, Inc., Japan. Triton X-100 was sourced from Merck & Co., Inc., USA. H_2_O_2_ was purchased from Guangzhou Chemical Reagent Factory. The RNA extraction kit was obtained from Seven Innovation (Beijing) Biotechnology Co., Ltd.

### Preparation of carriers and complexes

Mn-CDs were synthesized via a solvothermal method [16]. Specifically, 1.2167 g of L-Cys and 34.77 mg of MnO_2_ were dissolved in 10 mL of water, while 58 mg of NR was dissolved in 30 mL of absolute ethanol. The 2 solutions were combined and transferred into a 100-mL Teflon-lined autoclave, followed by reaction in a stainless-steel autoclave at 140 °C for 8 h. After natural cooling to room temperature, an orange-red suspension was obtained. The supernatant was then collected by centrifugation and filtered, and the filtrate was purified through rotary evaporation and dialysis. Finally, the purified product was dried in a freeze dryer to obtain Mn-CDs powder.

Twenty milligrams of PEI (10,000 Da) was dissolved in 50 mL of PBS; 10 mg of HA, 26 mg of EDC, and 16 mg of NHS were dissolved in 10 mL of PBS. The 2 solutions were ultrasonically mixed and then kept in the dark for 3 h to prepare the PEI-HA solution. Subsequently, 100 mg of Mn-CDs powder, 250 mg of EDC, and 100 mg of NHS were dissolved in 25 mL of PBS. After standing for 1 h, this mixture was combined with the PEI-HA solution and magnetically stirred at a constant speed in a constant-temperature water bath at 60 °C for 5 h to obtain the Mn-CDs-PEI-HA solution. The solution was then centrifuged and filtered, after which the filtrate was dialyzed and freeze-dried to obtain Mn-CDs-PEI-HA powder.

Finally, 40 μg of GP73-siRNA and varying masses of Mn-CDs-PEI-HA (80, 160, 320, 400, 600, 800, 1,000, 1,400, and 1,800 μg) were separately dissolved in DEPC water, followed by filtration through a 0.22-μm sterile filter membrane. The GP73-siRNA and Mn-CDs-PEI-HA solutions were then mixed on ice at different mass ratios (1:2, 1:4, 1:8, 1:10, 1:15, 1:20, 1:25, 1:30, 1:35, and 1:45) and allowed to stand for 45 min to prepare Mn-CDs-PEI-HA/GP73-siRNA complex solutions at different proportions.

### Characterization

The morphology, size distribution, and microstructure of the samples were observed using a high-resolution transmission electron microscope (JEOL JEM-2100F, Japan). Raman spectra were recorded by a Raman spectrometer (Horiba LabRAM HR Evolution, Japan). The functional groups of the samples were determined using a Fourier-transform infrared spectrometer (TENSOR27, Germany). The elemental composition of the samples was measured using x-ray photoelectron spectrometry (XPS; Thermo Scientific ESCALAB 250Xi, USA). Ultraviolet–visible (UV–Vis) absorption spectra and FL spectra were measured using an ultraviolet spectrophotometer (756 MC, Shanghai, China) and a fluorescence spectrometer (F-4700, Japan; light source: 150-W ozone-free xenon lamp), respectively. The content of Mn^2+^ was tested using an inductively coupled plasma–optical emission spectrometer (Agilent 5110, USA). The amount of O_2_ generated was measured with a portable dissolved oxygen meter (Oxi 7310; WTW inoLab, Germany). Zeta potential was measured via dynamic light scattering (Nano-ZS90; Malvern, UK). Cellular imaging was performed on a confocal laser scanning microscope (CLSM). In vivo mouse imaging and tissue imaging were captured using a small-animal 3D in vivo imaging system (PerkinElmer, Germany). Mouse magnetic resonance imaging (MRI) was conducted with a small-animal MRI system (Aspect Imaging, Israel). Agarose gel electrophoresis results were recorded using a gel imaging system (Gelview 6000M, China).

### Cell culture

Huh-7 and Hepa1-6 cells were cultured in high-glucose DMEM, while THLE-2 cells were maintained in 1640 medium, which contained 10% FBS and 1% penicillin–streptomycin. The cells were incubated at 37 °C in a humidified atmosphere containing 5% carbon dioxide (CO_2_) using a Thermo Fisher incubator to simulate natural physiological conditions and promote cell proliferation.

### O_2_ generation capacity

#### Detection of CAT-like activity

Under conditions of pH 6.5 and 7.4, 100 μL of H_2_O_2_ (100 mM) was added to PBS solutions containing identical concentrations of Mn-CDs and Mn-CDs-PEI-HA (200 μg/mL). A portable dissolved O_2_ meter was used to monitor the O_2_ generation every 10 s for a duration of 2,500 s to evaluate their catalytic O_2_ production capacity.

#### Detection of intracellular O_2_ level

Since the FL intensity of RDPP can be quenched by O_2_, it was employed to detect intracellular O_2_ generation. Huh-7 cells in good condition were uniformly seeded into 6-well plates at a density of 1 × 10^5^ cells/mL and incubated for 24 h in a constant-temperature incubator at 37 °C with 5% CO_2_. Subsequently, the cells were placed in a hypoxia chamber for an additional 12 h of culture. Mn-CDs and Mn-CDs-PEI-HA (200 μg/mL) were respectively dissolved in high-glucose DMEM containing RDPP (10 μg/mL), followed by further incubation for 6 h. The FL intensity of RDPP was then measured within the range of 508 to 750 nm (*λ*_ex_ = 488 nm), and images were captured.

### Biocompatibility

#### Hemocompatibility

Fresh healthy mouse blood was collected into anticoagulant tubes to prevent coagulation, diluted with PBS, and centrifuged at 1,000 rpm for 5 min to isolate red blood cells (RBCs) from the whole blood. The RBCs were repeatedly washed with PBS until the supernatant became clear, and a 2% RBC suspension was prepared with PBS. Subsequently, the RBC suspension was mixed with equal volumes of Mn-CDs solution (experimental group), Mn-CDs-PEI-HA solution (experimental group), PBS (negative control group), and Triton X-100 (positive control group), with the final concentrations in the experimental groups being 3.125, 6.25, 12.5, 25, 50, 100, and 200 μg/mL, respectively. After incubating the mixtures at 37 °C for 3 h, they were centrifuged at 1,500 rpm for 5 min. Finally, the absorbance of the supernatant at 575 nm (*I*) was measured. The hemolysis rate of the experimental groups was calculated using the following formula:

$$\text{Hemolysis}\;\left(\%\right)=\frac{\left(I_{\text{sample}}-I_{\text{negative control}}\right)}{\left(I_{\text{positive control}}-I_{\text{negative control}}\right)}\times 100\%$$ (1)

Here, *I* represents the absorbance at 575 nm. A hemolysis rate exceeding 5% is considered indicative of hemolytic activity in the experimental groups.

#### In vivo biocompatibility

To evaluate the biocompatibility of Mn-CDs and Mn-CDs-PEI-HA, healthy 6-week-old BALB/c mice were randomly divided into 3 groups and administered 100 μL of normal saline, Mn-CDs, and Mn-CDs-PEI-HA (10 mg/kg), respectively, via tail vein injection. The body weight of the mice in all 3 groups was monitored every 3 d for 28 d to assess potential systemic toxicity. Blood and major organs were collected at 0, 1, 14, and 28 d for analysis of complete blood count, blood biochemistry, and histopathological examination of tissues including the heart, liver, spleen, kidneys, and lungs.

### GA co-localization imaging

Huh-7 cells were seeded in confocal dishes at a density of 4 × 10^4^/mL and cultured for 24 h, followed cultured under starvation conditions for 1 h. The cells were then incubated with 200 μg/mL Mn-CDs-PEI-HA at 37 °C for 4 h. After washing 3 times with PBS, the cells were stained with a commercial green FL GA probe at 37 °C for 30 min. Finally, the cells were washed 3 times with PBS, fixed with paraformaldehyde for 10 min, and imaged using laser scanning confocal microscopy with an excitation wavelength of 552 nm and an emission wavelength of 625 nm.

### Targeting dual-modality imaging

FL imaging: Tumor-bearing mice (*n* = 3) were housed under a 12-h light/dark cycle at an ambient temperature of 20 to 26 °C and relative humidity of 40% to 70%. After intravenous injection of Mn-CDs-PEI-HA (10 mg/kg), in vivo FL signals were collected at different time points (0, 2, 4, 6, 12, and 24 h) using a small-animal in vivo imaging system (PerkinElmer, Germany) with an excitation wavelength of 500 nm and an emission wavelength of 620 nm. Subsequently, the tumor-bearing nude mice were euthanized and dissected to harvest major organs including the heart, liver, spleen, lungs, kidneys, and tumors for ex vivo FL imaging. Finally, the data were analyzed using Living Image software (v4.3.1).

MRI: Tumor-bearing mice (*n* = 3) were subjected to MRI using a small-animal MRI system before and 6 h after intravenous injection of Mn-CDs-PEI-HA (10 mg/kg). T1 rapid acquisition with relaxation enhancement and T1 mapping rapid acquisition with relaxation enhancement axial images were acquired using an Aspect M3 system with the following parameters: field of view, 3.0 × 6.0 cm; slice thickness, 1 mm; slice gap, 1 mm; and repetition time, 300 ms.

### Gene-silencing efficiency

Total RNA was extracted using the SM130-02 kit (SEVEN Biotech) according to the manufacturer’s instructions and reverse-transcribed into cDNA using the SM143-01 kit. Finally, GP73-mRNA expression was quantified by quantitative real-time polymerase chain reaction (qRT-PCR) with a 7500 Real-Time PCR System (Thermo Scientific, MA, USA) to determine the silencing efficiency of the GP73-siRNA sequence against GP73-mRNA.

### Gene stability within the complex

GelRed nucleic acid dye was used to perform electrophoresis analysis on 1% agarose gel in Tris–acetate–EDTA buffer at 180 mA and 120 V for 20 min to examine the band migration of GP73-siRNA and Mn-CDs-PEI-HA at different mass ratios of Mn-CDs-PEI-HA/GP73-siRNA, and the binding capacity between the carrier and the gene.

To evaluate the stability of Mn-CDs-PEI-HA/GP73-siRNA in serum, it was incubated with 10% FBS in a 37 °C water bath for 0, 6, 12, 24, 48, and 72 h, respectively, followed by electrophoresis. The electrophoresis results were recorded using a gel imaging system.

### In vitro antitumor efficacy

#### CCK-8 assay

Huh-7 and THLE-2 cells were seeded in 96-well plates at a density of 1 × 10^5^ cells/mL and incubated for 24 h under normoxic (21% O_2_) or hypoxic (1% O_2_) conditions at 37 °C. The medium was then replaced with fresh medium containing different concentrations of Mn-CDs, Mn-CDs-PEI-HA, GP73-siRNA, and Mn-CDs-PEI-HA/GP73-siRNA, and the cells were treated under different conditions for 24 h. Subsequently, the medium was replaced with complete medium containing 10 μL of CCK-8 solution, followed by incubation at 37 °C for 2 h. The absorbance of each well at 450 nm was measured using a microplate reader to evaluate the inhibitory effects on cell proliferation across different groups.

#### Live/dead cell staining assay

Huh-7 cells were incubated with complete medium (control group), Mn-CDs, Mn-CDs-PEI-HA, GP73-siRNA, and Mn-CDs-PEI-HA/GP73-siRNA under normoxic and hypoxic conditions for 24 h, respectively, and then washed 2 times with PBS. The cells were then stained with Calcein-AM and propidium iodide (PI) solution for 20 min, washed 3 times with PBS, and imaged using FL microscopy to observe live and dead cells.

#### Cell invasion assay

The cell invasion assay was performed using permeable chambers. Firstly, serum-free blank medium was mixed with Matrigel at volume ratio of 8:1, and 60 μL of the mixture was added to each upper chamber to form a polymerized thin film, which was incubated for 3 h before removing the remaining medium from the upper chamber. Subsequently, Huh-7 cells (1 × 10^6^ cells/mL), starved for 24 h, were resuspended in complete medium (control group), Mn-CDs, Mn-CDs-PEI-HA, GP73-siRNA, and Mn-CDs-PEI-HA/GP73-siRNA mixtures and seeded into the upper chambers. After incubation under normoxic or hypoxic conditions for 48 h, the upper chambers were washed 3 times with PBS, fixed with 4% paraformaldehyde for 12 min, and washed again 3 times with PBS. The cells were then stained with 500 μL of crystal violet solution for 10 min and washed 3 times with PBS to remove excess dye. The inner surface of the upper membrane was gently wiped with a cotton swab. Finally, cells in 3 predefined areas of the upper membrane were imaged and counted using an inverted FL microscope to evaluate the effect of different treatments on cell invasion.

### In vivo antitumor efficacy

All animal experiments were purchased from Beijing Vital River Laboratory Animal Technology Co., Ltd. and were used with the approval of the Animal Protection Committee of Shanxi Province Cancer Hospital (ethics number: DWLL2025018). Hepa1-6 cells (1 × 10^6^ cells/mL) were subcutaneously injected into the right flank of BALB/c nude mice and C57 mice, respectively. When the tumor volume reached approximately 100 mm^3^, the mice were divided into 5 groups (*n* = 5): (a) saline, (b) Mn-CDs, (c) Mn-CDs-PEI-HA, (d) GP73-siRNA, and (e) Mn-CDs-PEI-HA/GP73-siRNA. Thereafter, tumor-bearing mice were intravenously injected every 3 d with 100 μL of saline, Mn-CDs, Mn-CDs-PEI-HA, GP73-siRNA, and Mn-CDs-PEI-HA/GP73-siRNA (10 mg/kg). Tumor volume and body weight were recorded every 2 d using a digital caliper and a laboratory balance. Tumor volume was calculated as (width^2^ × length)/2, where “width” and “length” represent the shortest and longest dimensions of the tumor, respectively. After 14 d of treatment, the mice were euthanized, and tumors and major organs were collected for hematoxylin and eosin (H&E) staining to evaluate histopathological toxicity.

### Statistical analysis

Data are presented as mean ± standard deviation (SD). Comparisons between 2 groups were performed using paired Student *t* test, and statistical significance was assessed by one-way analysis of variance. A *P* value <0.05 was considered statistically significant, with *P* <0.001 denoted as ***. All analyses were conducted using GraphPad Prism 9.

## Results

### Construction and characterization of the carrier

As shown in Fig. 1, Mn-CDs were synthesized via a one-pot solvothermal method using L-Cys, MnO_2_, and NR as precursors. Subsequently, PEI with high gene transfection efficiency [16] was conjugated with HA, which targets CD44 receptors on tumor surfaces [17], to form PEI-HA. Then, PEI-HA was modified onto the surface of Mn-CDs to synthesize Mn-CDs-PEI-HA. The morphology, structure, and optical properties of Mn-CDs and Mn-CDs-PEI-HA were characterized.

Transmission electron microscopy (TEM) images revealed that both Mn-CDs and Mn-CDs-PEI-HA exhibited spherical morphology, were well dispersed without significant aggregation, and had particle sizes of 1.82 and 3.56 nm, respectively. The increased particle size of Mn-CDs-PEI-HA compared with Mn-CDs indicates that PEI-HA was grafted onto the Mn-CDs surface (Fig. 2A and B). Energy-dispersive spectroscopy confirmed that 9.27 wt.% of manganese is contained in Mn-CDs. Elemental mapping images (Fig. 2D) demonstrated that C, N, O, S, and Mn elements were uniformly distributed on the surface of Mn-CDs [18]. Furthermore, high-resolution TEM images revealed distinct lattice fringes in Mn-CDs and Mn-CDs-PEI-HA with an interplanar spacing of 0.21 nm, corresponding to the (001) crystal plane of graphitic carbon, indicating a certain degree of graphitization in both Mn-CDs and Mn-CDs-PEI-HA [19].

**Fig. 2.**
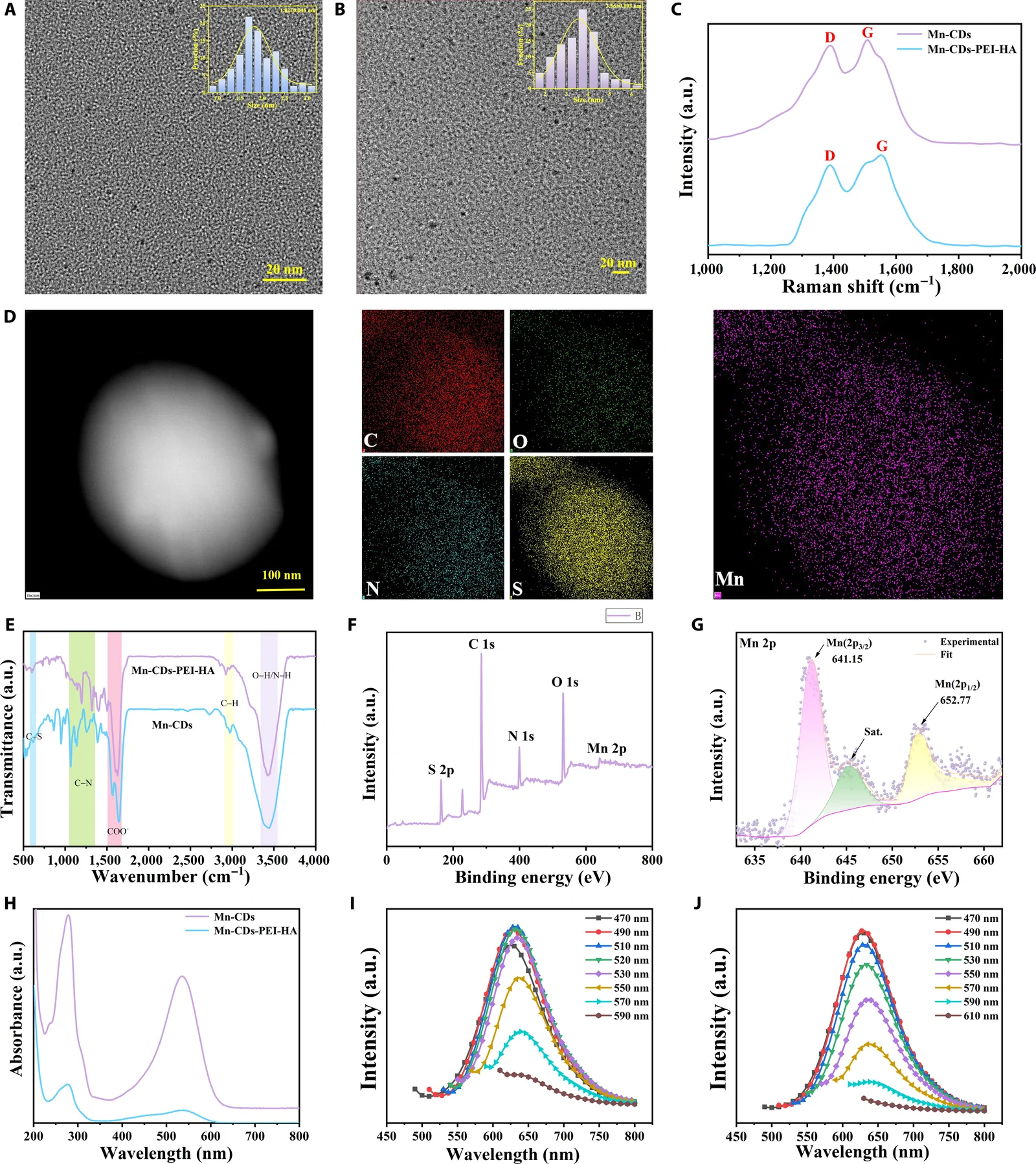
TEM images of (A) Mn-CDs and (B) Mn-CDs-PEI-HA; (C) Raman spectra of Mn-CDs and Mn-CDs-PEI-HA; (D) elemental mapping of Mn-CDs; (E) Fourier-transform infrared spectra of Mn-CDs and Mn-CDs-PEI-HA; (F) x-ray photoelectron spectrometry (XPS) survey spectrum and (G) Mn 2p XPS spectrum of Mn-CDs; (H) normalized UV–Vis spectra of Mn-CDs and Mn-CDs-PEI-HA; emission spectra of (I) Mn-CDs and (J) Mn-CDs-PEI-HA.

The Raman spectra of Mn-CDs (Fig. 2C) exhibited characteristic peaks at 1,551 and 1,389 cm^−1^, corresponding to the G-band and D-band, respectively. The intensity ratio of G-band to D-band (*I*_G_/*I*_D_) was 0.90, indicating a certain degree of graphitization [20]. The Raman spectra of Mn-CDs-PEI-HA showed characteristic peaks at 1,509 and 1,391 cm^−1^, assigned to the G-band and D-band, respectively, with an *I*_G_/*I*_D_ of 0.92. The subtle increased ratio compared with Mn-CDs may result from altered molecular structure or chemical environment originated from the covalent linkage of PEI-HA to Mn-CDs [21]. Fourier-transform infrared analysis (Fig. 2E) revealed that both Mn-CDs and Mn-CDs-PEI-HA possess abundant surface functional groups, including O–H/N–H (3,300 to 3,500 cm^−1^), C–H (2,800 to 3,000 cm^−1^) [18], COO– (1,550 to 1,610 cm^−1^) [22], C–N (1,000 to 1,350 cm^−1^), and C–S (600 to 620 cm^−1^) [23]. The absorption band at 610 cm^−1^ was attributed to the stretching vibration of Mn–O, confirming Mn doping in both Mn-CDs and Mn-CDs-PEI-HA [24]. Notably, compared with Mn-CDs, Mn-CDs-PEI-HA displayed characteristic absorption bands at 1,627 and 2,922 cm^−1^ [24], indicating the presence of the amide I band (C=O) [25], suggesting that HA was modified onto the surface of Mn-CDs. Additionally, the asymmetric C–N absorption peak between 1,195 and 1,373 cm^−1^ indicates that PEI was grafted onto the surface of Mn-CDs, collectively demonstrating that PEI-HA was modified onto the surface of Mn-CDs.

XPS analysis was further employed to investigate the elemental composition and chemical structure of the samples. The XPS survey spectrum (Fig. 2F) revealed the presence of S, C, N, O, and Mn elements in Mn-CDs. The Mn 2p spectrum of Mn-CDs (Fig. 2G) exhibited 2 main peaks at 641.15 and 652.77 eV, along with a characteristic satellite peak at 645.35 eV, corresponding to Mn 2p_1/2_, Mn 2p_3/2_, and satellite, respectively. The high-resolution Mn 3s spectrum of Mn-CDs (Fig. S1A) showed a Δ*E* of 5.96 eV between the 2 peaks, indicating that Mn was doped into the Mn-CDs in the form of Mn^2+^ [26].

UV–Vis spectra showed distinct absorption peaks at wavelengths of 279 and 536 nm (Fig. 2H). After modification with PEI-HA, the absorption peak at 536 nm broadened [24]. Mn-CDs and Mn-CDs-PEI-HA exhibited weak excitation dependence. The optimal excitation and emission wavelengths of Mn-CDs are 510 and 630 nm, respectively (Fig. 2I), while the optimal emission and excitation wavelengths of Mn-CDs-PEI-HA are 490 and 642 nm, respectively (Fig. 2J), indicating that the modification with PEI-HA promoted the red shift in the emission wavelength of Mn-CDs-PEI-HA [27,28]. Furthermore, the absorbance of Mn-CD nanoenzyme at 536 nm remained basically unchanged over a period of 7 d, indicating that the nanoenzyme have good stability (Fig. S1D).

### O_2_ generation capacity of the carrier

The release of Mn^2+^ from Mn-CDs facilitates the subsequent catalytic decomposition of endogenous H_2_O_2_ to generate O_2_, thereby ameliorating the hypoxic microenvironment and inhibiting proliferation of HCC. Firstly, the Mn^2+^ release capability of Mn-CDs was investigated in buffer solutions at different pH values, as shown in Fig. 3A. In an acidic environment (pH = 6.5), Mn^2+^ was gradually released from Mn-CDs, achieving a cumulative release rate exceeding 70%. In contrast, under neutral conditions (pH = 7.4), the Mn^2+^ release was only approximately 15%, indicating that Mn-CDs can release a substantial amount of Mn^2+^ in the acidic tumor microenvironment [29,30].

**Fig. 3.**
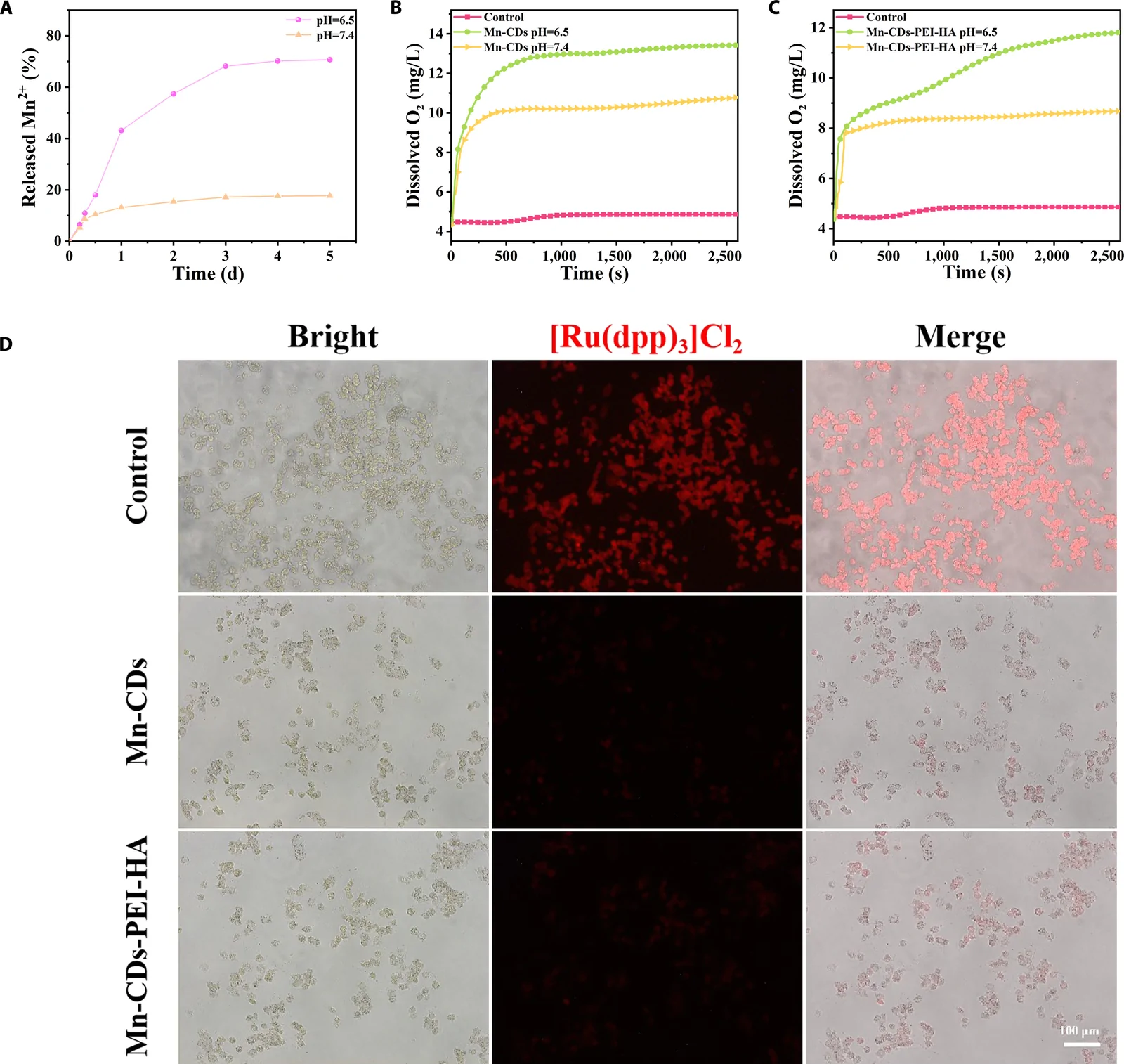
(A) Mn^2+^ release profile of Mn-CDs; dissolved O_2_ curves of (B) Mn-CDs and (C) Mn-CDs-PEI-HA; (D) fluorescence (FL) images of in vitro cellular O_2_ generation by Mn-CDs and Mn-CDs-PEI-HA.

Following the release of Mn^2+^, it can catalyze the decomposition of H_2_O_2_, to generate O_2_, (as shown in Eqs. (2) and (3)) [29–32], thereby ameliorating the hypoxic tumor microenvironment.

$${\mathrm{Mn}}^{2+}+H_{2}O_{2}\to {\text{2H}}^{+}+{\mathrm{MnO}}_{2}$$ (2)

$${\mathrm{MnO}}_{2}+H_{2}O_{2}+{\text{2H}}^{+}\to {\mathrm{Mn}}^{2+}+{\text{2H}}_{2}O+O_{2}↑$$ (3)

To further investigate whether Mn-CDs and Mn-CDs-PEI-HA possess CAT-like activity, O_2_ generation by Mn-CDs and Mn-CDs-PEI-HA (200 μg/mL) in the presence of H_2_O_2_ solution (100 μM) was examined under different pH conditions (6.5 and 7.4), as shown in Fig. 3B and C. Both materials produced more O_2_ under acidic conditions (pH = 6.5, 12 mg/L within 10 min) than under neutral conditions (pH = 7.4), which is attributed to the accelerated reaction rate of Eq. (3) at lower pH, resulting in faster O_2_ generation under acidic conditions compared with neutral conditions [31].

Finally, the RDPP FL probe was used to investigate the O_2_ generation by Mn-CDs and Mn-CDs-PEI-HA in cells in vitro. RDPP is an O_2_-sensitive indicator probe whose FL is quenched upon O_2_ generation in the environment, thereby reflecting changes in O_2_ levels [32]. The results in Fig. 3D and Fig. S1E show that after incubating Huh-7 cells with Mn-CDs and Mn-CDs-PEI-HA for 6 h, the control group exhibited strong RDPP FL signals, indicating a hypoxic state. In contrast, cells incubated with Mn-CDs and Mn-CDs-PEI-HA showed significantly quenched RDPP FL intensity, demonstrating intracellular O_2_ generation and alleviation of tumor-cell hypoxia, indicating that both Mn-CDs and Mn-CDs-PEI-HA can catalyze the decomposition of excess H_2_O_2_ in hypoxic tumors to produce O_2_, thereby ameliorating the tumor hypoxic microenvironment (the process shown in Fig. S1F occurs within the cells) [33,34]. Moreover, the signal-to-noise ratios for the control, Mn-CDs, and Mn-CDs-PEI-HA groups are 16.5, 4.6, and 6.3, respectively (Fig. S1G). It can be concluded that the FL signals in both the Mn-CDs and Mn-CDs-PEI-HA groups were significantly lower than those in the control group, suggesting that both Mn-CDs and Mn-CDs-PEI-HA can alleviate cellular hypoxia, and Mn-CDs is superior to Mn-CDs-PEI-HA. In summary, although the HCC microenvironment (hypoxia, glutathione, and H_2_O_2_) partially reduced the high-valence Mn^4+^ sites to Mn^2+^, the CAT-like activity of Mn-CDs and Mn-CDs-PEI-HA were not completely quenched. This is attributed to the simultaneous presence of endogenous H_2_O_2_ and acidic pH, which continuously re-oxidize Mn^2+^ back to Mn^4+^, establishing a dynamic catalytic cycle that guarantees sustained O_2_ generation. This clearly indicates that Mn-CDs and Mn-CDs-PEI-HA not only improves the tumor hypoxic microenvironment but also enhances therapeutic efficacy.

### Biocompatibility of the carrier

Evaluating the biocompatibility of Mn-CDs and Mn-CDs-PEI-HA is essential for their biomedical applications. Firstly, hemolysis assay was conducted to determine the extent of RBC rupture upon contact with Mn-CDs and Mn-CDs-PEI-HA [35,36]. As shown in Fig. 4A, even at a concentration of 200 μg/mL, Mn-CDs and Mn-CDs-PEI-HA did not cause RBC rupture, with hemolysis rates of 3.3% and 2.9%, respectively. Both are below 5% (Fig. 4B), indicating excellent hemocompatibility and no damage to RBCs [26,37]. In vivo biocompatibility results (Fig. 4D) in mice showed that, compared with the control group, the experimental groups exhibited no significant increase in serum levels of alanine aminotransferase (ALT), aspartate aminotransferase (AST), urea nitrogen (UREA), or creatinine (CREA). Moreover, no notable changes in body weight or tissue damage were observed (Fig. 4C and E), demonstrating that the prepared carriers Mn-CDs and Mn-CDs-PEI-HA possess favorable biocompatibility and are suitable for subsequent in vivo therapy [38,39].

**Fig. 4.**
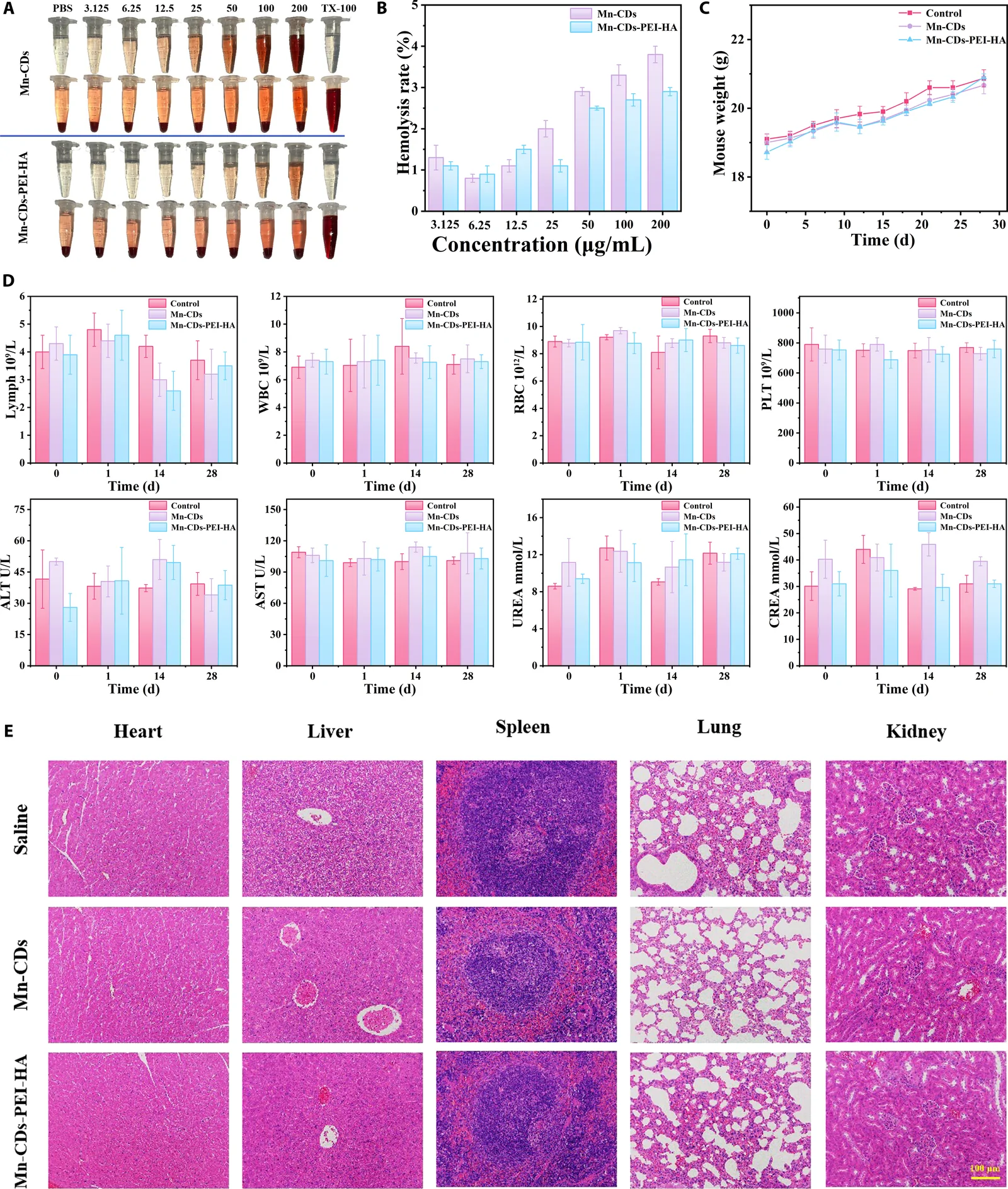
(A) Hemolysis assay images and (B) hemolysis rate statistics of Mn-CDs and Mn-CDs-PEI-HA; (C) body weight change curves, (D) complete blood count and blood biochemical analysis at different time, and (E) H&E staining sections of mice after tail vein injection of Mn-CDs and Mn-CDs-PEI-HA. PBS, phosphate-buffered saline; TX-100, Triton X-100; WBC, white blood cell; RBC, red blood cell; PLT, platelet; ALT, alanine aminotransferase; AST, aspartate aminotransferase; UREA, urea nitrogen; CREA, creatinine.

To further investigate the in vivo distribution of Mn-CDs and Mn-CDs-PEI-HA, they were injected into the mice via tail vein (10 mg/kg), and the FL signals in live animals and major isolated organs were observed at 0, 2, 4, 6, 12, and 24 h using a small-animal in vivo imaging system. As shown in Fig. S2A and B, the majority of Mn-CDs and Mn-CDs-PEI-HA accumulated in the liver, followed by the kidneys, with minimal amounts observed in the lungs, spleen, and heart. The accumulation of Mn-CDs and Mn-CDs-PEI-HA in mice peaked at 6 h post-injection and subsequently declined, with near-complete clearance from the body by 24 h. These results indicate that Mn-CDs and Mn-CDs-PEI-HA possess favorable biocompatibility [20] and can be further applied for in vivo cancer monitoring and therapy.

### Dual-modality imaging and dual-targeting properties of the carrier

The GA targeting capability of Mn-CDs-PEI-HA was evaluated through FL co-localization experiments. As shown in Fig. 5A, the red FL signal from Mn-CDs-PEI-HA overlapped well with the green channel of the commercial GA FL dye (NBD), and the Pearson correlation coefficient is 0.84, indicating significant GA targeting ability [40,41] and FL imaging performance. To assess the in vivo tumor specificity and intratumoral permeability of Mn-CDs-PEI-HA [42], a subcutaneous tumor model was established, and Mn-CDs-PEI-HA (10 mg/kg) was administered via tail vein injection. In vivo FL imaging of small animals (Fig. 5B) revealed that the FL signal of Mn-CDs-PEI-HA began to accumulate in the tumor region after 2 h, gradually increasing over time, with the highest accumulation observed at 6 h. By 24 h, the FL signal had largely diminished, indicating near-complete clearance of Mn-CDs-PEI-HA from the body. Furthermore, ex vivo FL imaging of major organs (heart, liver, spleen, lungs, and kidneys) and tumor tissue (Fig. S2B) was performed to examine the biodistribution of Mn-CDs-PEI-HA. The results showed that the FL intensity in the tumor tissue gradually increased from 0 to 24 h and then progressively decreased (Fig. S3B). After euthanasia and dissection, the liver, lungs, and kidneys exhibited FL signals that decreased over time, indicating their active involvement in the metabolism of Mn-CDs-PEI-HA within 24 h post-injection. These findings are consistent with the in vivo FL imaging results (Fig. S2B). The biodistribution of Mn-CDs-PEI-HA in tumor-bearing mice further confirmed satisfactory tumor accumulation, demonstrating its dual-targeting capability for the GA and tumors, as well as their promising FL imaging potential [35,43].

**Fig. 5.**
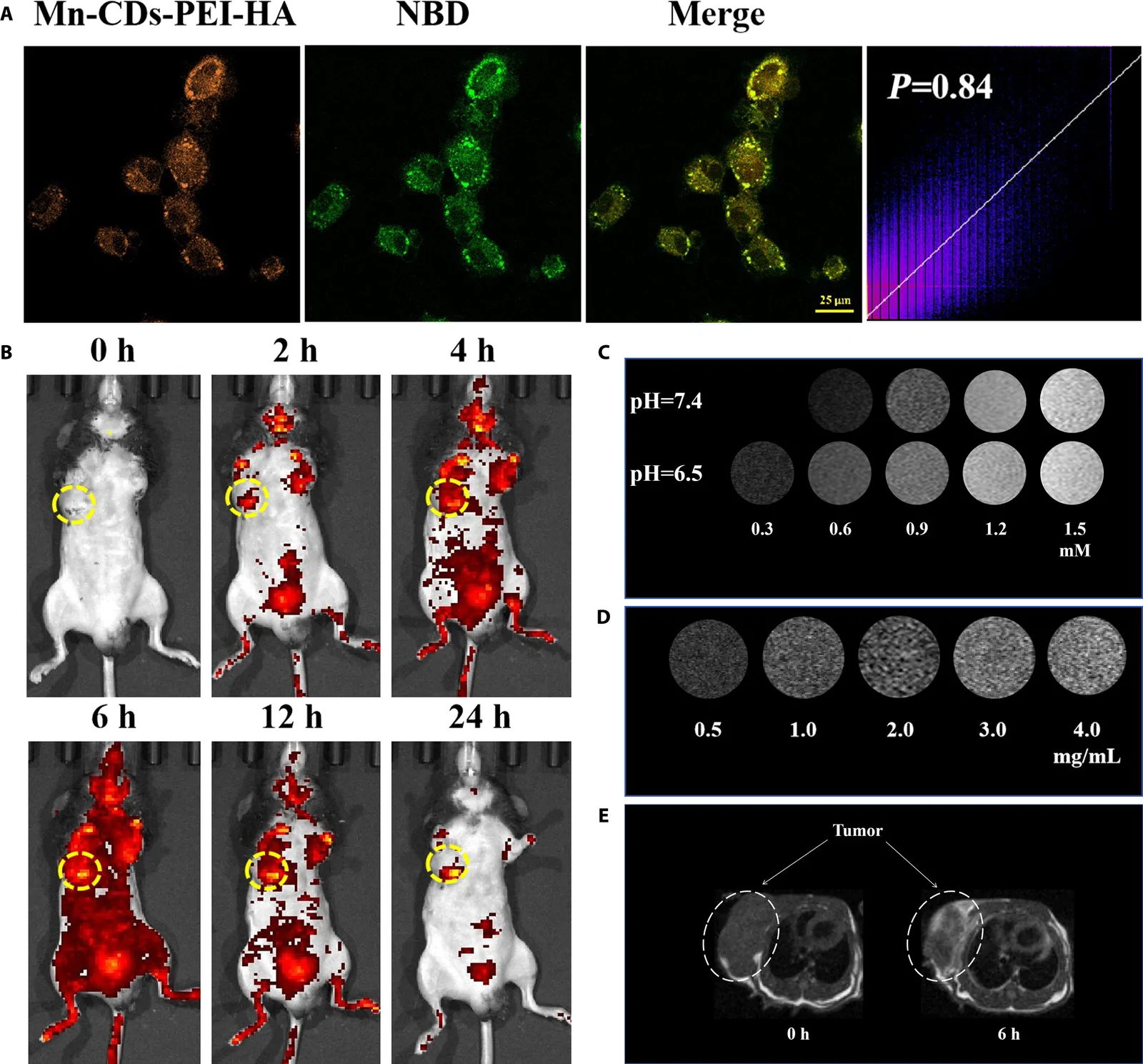
(A) FL co-localization images of Mn-CDs-PEI-HA in the Huh-7 cells; (B) in vivo FL images of mice at different times after tail vein injection of Mn-CDs-PEI-HA; (C) MR images of Mn-CDs solutions under different pH conditions; (D) MR images of Huh-7 cells incubated with different concentrations of Mn-CDs; (E) MR images of mice 6 h after tail vein injection of Mn-CDs-PEI-HA. NBD, *N*-(NBD-aminohexanoyl) D-erythro-sphingosine.

Owing to the high paramagnetism and low biotoxicity of Mn^2+^, Mn-CDs can serve as MRI agents. As shown in the MRIs (Fig. 5C), the MR signal intensified with increasing Mn^2+^ concentration. Furthermore, under acidic conditions, Mn-CDs exhibited stronger MR signals, attributable to the release of paramagnetic Mn^2+^ from Mn-CDs in an acidic environment (Fig. 2B) [44]. To further investigate the in vitro cellular MRI capability of Mn-CDs, Huh-7 cells were incubated with Mn-CDs for 4 h and subsequently subjected to MRI. The results in Fig. 5D demonstrate that cells treated with Mn-CDs displayed bright MR signals, with the signal intensity positively correlated with the concentration of Mn-CDs, confirming their potential as MRI agents. To evaluate the in vivo MRI performance and targeting ability of Mn-CDs-PEI-HA, T_1_-weighted MRI was performed on tumor-bearing mice 6 h after injection of Mn-CDs-PEI-HA (10 mg/kg). As shown in Fig. 5E, a significant enhancement of the T_1_-weighted signal was observed in the tumor region, indicating excellent in vivo MRI capability of Mn-CDs-PEI-HA for tumor tissues [43]. These results collectively demonstrate that Mn-CDs-PEI-HA possesses favorable dual-modality imaging capabilities and exhibits dual-targeting properties toward both the GA and tumor tissues.

### Construction of the complex and their gene stability

Initially, 3 synthesized therapeutic genes GP73-siRNA/241, GP73-siRNA/671, and GP73-siRNA/1274 were screened by RT-qPCR to compare their effects on GP73-mRNA expression. As shown in Fig. S4, GP73-siRNA/1274 exhibited the strongest inhibitory effect on GP73-mRNA. Therefore, subsequent experiments employed Mn-CDs-PEI-HA to load GP73-siRNA/1274 for the construction of the complex.

Subsequently, the interaction between Mn-CDs-PEI-HA and GP73-siRNA was investigated through agarose gel electrophoresis experiments. GP73-siRNA and Mn-CDs-PEI-HA were mixed at different mass ratios in DEPC water and then verified using 1% agarose gel electrophoresis [13,16]. As shown in Fig. 6A, free siRNA, being negatively charged, migrated toward the positive electrode, resulting in a bright migration band in the gel. When the mass ratio of GP73-siRNA to Mn-CDs-PEI-HA was 1:5, a bright migration band was still observed. However, when the ratio increased to 1:20, the siRNA migration band disappeared, indicating complete retardation of the complexed siRNA toward the positive electrode and demonstrating full loading of GP73-siRNA onto the surface of Mn-CDs-PEI-HA. These results confirm that a stable complex is formed via electrostatic interactions between GP73-siRNA and Mn-CDs-PEI-HA at a mass ratio of 1:20. The zeta potential of free GP73-siRNA was −14.7 mV, whereas Mn-CDs-PEI-HA/GP73-siRNA (the mass ratio of GP73-siRNA and Mn-CDs-PEI-HA is 1:20) exhibited a positive surface charge of +4.43 mV (Fig. 6B). Furthermore, TEM characterization revealed an increased size of 12.84 ± 0.17 nm for Mn-CDs-PEI-HA/GP73-siRNA compared with Mn-CDs-PEI-HA (Fig. 6C), confirming the GP73-siRNA was loaded onto the surface of Mn-CDs-PEI-HA.

**Fig. 6.**
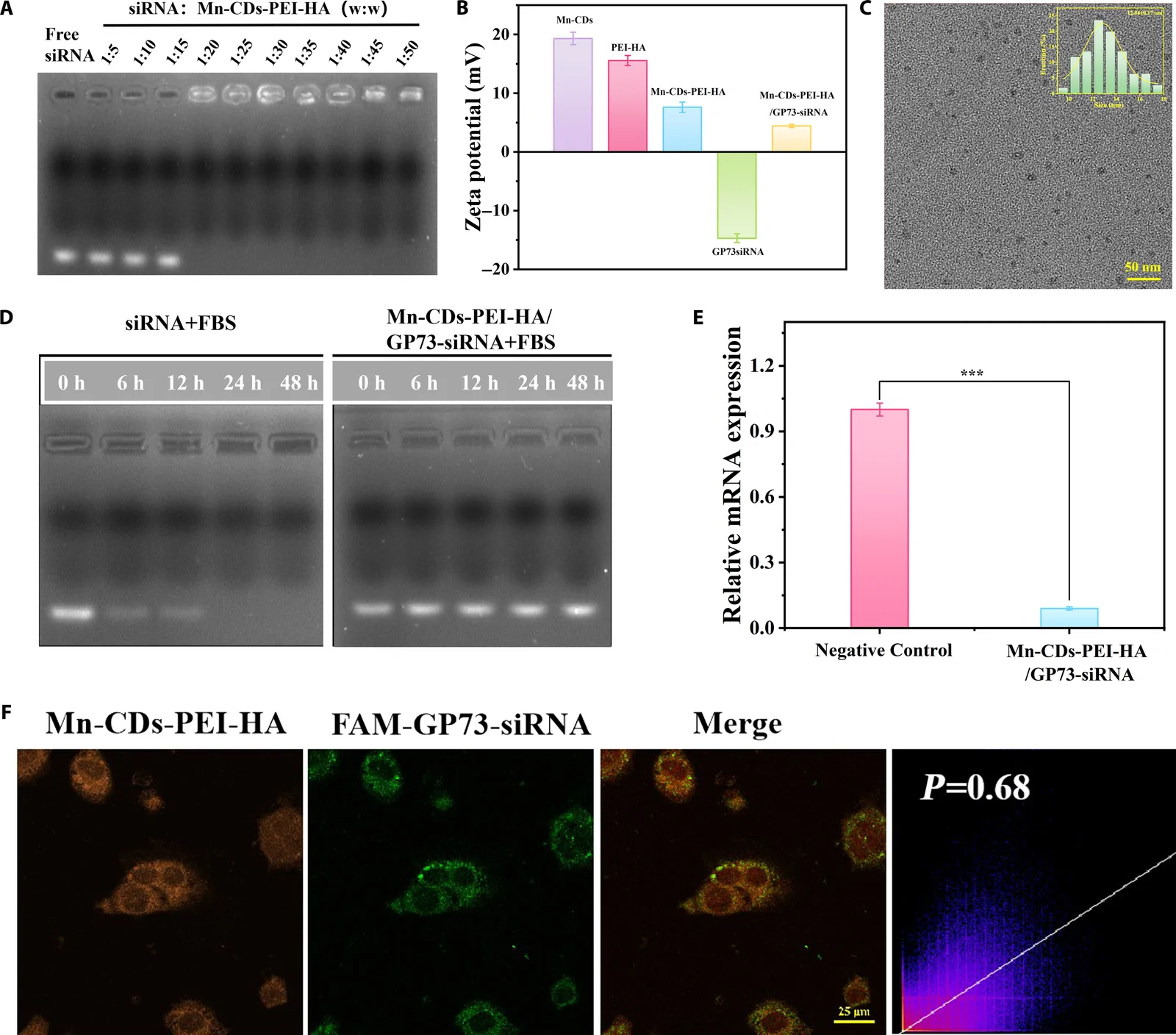
(A) Agarose gel electrophoresis of Mn-CDs-PEI-HA/GP73-siRNA obtained at different mass ratios; (B) zeta potentials of Mn-CDs, PEI-HA, Mn-CDs-PEI-HA, GP73-siRNA, and Mn-CDs-PEI-HA/GP73-siRNA; (C) TEM image of Mn-CDs-PEI-HA/GP73-siRNA; (D) comparison of stability between free GP73-siRNA and Mn-CDs-PEI-HA/GP73-siRNA in FBS by agarose gel electrophoresis; (E) GP73-mRNA expression level in Huh-7 cells after treatment with Mn-CDs-PEI-HA/GP73-siRNA; (F) CLSM images of Mn-CDs-PEI-HA/FAM-GP73-siRNA in Huh-7 cells. Data are presented as mean ± SD.

Enzymes in the bloodstream serve as the first barrier for Mn-CDs-PEI-HA/GP73-siRNA upon entering the body [13,45]. In the rational design of nanoparticle-based drug delivery systems, achieving robust serum stability is crucial for balancing prolonged systemic circulation [45]. To validate the serum stability of Mn-CDs-PEI-HA/GP73-siRNA, the stability of both free GP73-siRNA and the Mn-CDs-PEI-HA/GP73-siRNA complex was assessed in 10% FBS at various time intervals (Fig. 6D). After incubation in FBS for 24 h, no intact free GP73-siRNA was detected, indicating the rapid degradation of GP73-siRNA under FBS conditions without the protection of a carrier. In contrast, in the Mn-CDs-PEI-HA/GP73-siRNA group, the GP73-siRNA band remained largely intact even after 48 h, demonstrating that Mn-CDs-PEI-HA can serve as an excellent carrier for GP73-siRNA delivery, effectively protecting it from enzymatic degradation in FBS.

Gene transfection efficiency is a critical indicator for evaluating the performance of gene delivery systems [45]. The silencing effect of GP73-mRNA in Huh-7 cells by Mn-CDs-PEI-HA/GP73-siRNA was verified via RT-qPCR. As shown in Fig. 6E, compared with the blank control group, after 24 h of transfection with GP73-siRNA delivered by Mn-CDs-PEI-HA, the silencing efficiency reached 91%, significantly reducing the GP73-mRNA expression level in Huh-7 cells (*P* < 0.001).

To further evaluate the cellular transfection capability of Mn-CDs-PEI-HA/GP73-siRNA, in vitro cellular imaging studies were performed on Huh-7 cells using a CLSM. As shown in Fig. 6F, Mn-CDs-PEI-HA exhibited orange-red emissive FL, while the FAM-labeled GP73-siRNA (FAM-GP73-siRNA) exhibits green FL, and the “Merge” image represents the overlay of the 2 FL channels. The images demonstrate that Mn-CDs-PEI-HA/GP73-siRNA is distributed within the cytoplasm, and the FL signals of Mn-CDs-PEI-HA and GP73-siRNA overlap with each other. The Pearson co-localization coefficient of 0.68 (>0.5) further indicates that Mn-CDs-PEI-HA can effectively deliver GP73-siRNA into cells. The above results demonstrate that Mn-CDs-PEI-HA/GP73-siRNA have favorable cellular internalization ability and Mn-CDs-PEI-HA can effectively carry GP73-siRNA into cells, enhance transfection efficiency, and consequently inhibit GP73-mRNA expression.

### In vitro antitumor activity of the complex

To further investigate the cytotoxicity and antitumor efficacy of the complex in vitro, the viability of THLE-2 (normal hepatocytes) and Huh-7 cells (HCC cells) was assessed using the CCK-8 assay, allowing evaluation of the toxicity and antitumor performance of Mn-CDs and Mn-CDs-PEI-HA under in vitro conditions. As shown in Fig. 7A and B, after THLE-2 cells were incubated with varying concentrations of Mn-CDs and Mn-CDs-PEI-HA for 24 h, the cell viability of THLE-2 remained above 80%, indicating minimal cytotoxic effects of the carriers on normal hepatocytes. Both Mn-CDs and Mn-CDs-PEI-HA have low toxicity. After Huh-7 cells were incubated with varying concentrations of Mn-CDs and Mn-CDs-PEI-HA for 24 h under normoxic conditions, the cell viability decreased as the concentration of Mn-CDs and Mn-CDs-PEI-HA increased. When the carrier concentration reached 200 μg/mL, the cell survival rates were approximately 50% and 60%, respectively, indicating an inhibitory effect on the proliferation of tumor cells. As shown in Fig. 7C and D, the cell viability under hypoxic conditions was consistently lower than that under normoxic conditions when treated with the same concentrations of Mn-CDs and Mn-CDs-PEI-HA. Specifically, the cell survival rates of Mn-CDs and Mn-CDs-PEI-HA group were approximately 40% and 50% under hypoxia conditions, respectively, demonstrating that both materials can ameliorate the tumor hypoxic microenvironment and thereby inhibit tumor growth. Furthermore, at equivalent concentrations, Mn-CDs exhibited a stronger inhibitory effect than Mn-CDs-PEI-HA, which can be attributed to the higher O_2_ production capacity of Mn-CDs compared to Mn-CDs-PEI-HA at the same concentration (Fig. S1H). To further validate the therapeutic efficacy of Mn-CDs-PEI-HA/GP73-siRNA, cell viability was assessed under both normoxic and hypoxic conditions. As shown in Fig. 7E and F, under hypoxic conditions, Mn-CDs, Mn-CDs-PEI-HA, and Mn-CDs-PEI-HA/GP73-siRNA all exhibited greater inhibitory toward Huh-7 cells compared to normoxic conditions, demonstrating that they can ameliorate the tumor hypoxic microenvironment and kill cancer cells. Moreover, under both normoxic and hypoxic conditions, Mn-CDs-PEI-HA/GP73-siRNA showed significantly enhanced inhibitory effects on Huh-7 cells compared with the Mn-CDs and Mn-CDs-PEI-HA groups, with inhibition rates of 70% and 85%, respectively, indicating that Mn-CDs-PEI-HA/GP73-siRNA inhibits cell proliferation by exerting the dual effects of alleviating hypoxia and mediating gene silencing. As shown in Fig. 7G and H, the cytotoxicity of Mn-CDs-PEI-HA/GP73-siRNA against Huh-7 cells increased with concentration under both normoxic and hypoxic conditions. After incubation with Mn-CDs-PEI-HA/GP73-siRNA (200 μg/mL) for 24 h, the Huh-7 cells’ viability was only approximately 15% under hypoxic conditions, compared with about 30% under normoxic conditions. These results indicate that Mn-CDs-PEI-HA/GP73-siRNA achieved tumor-cell killing through the dual mechanisms of ameliorating the hypoxic microenvironment and mediating gene-silencing therapy.

**Fig. 7.**
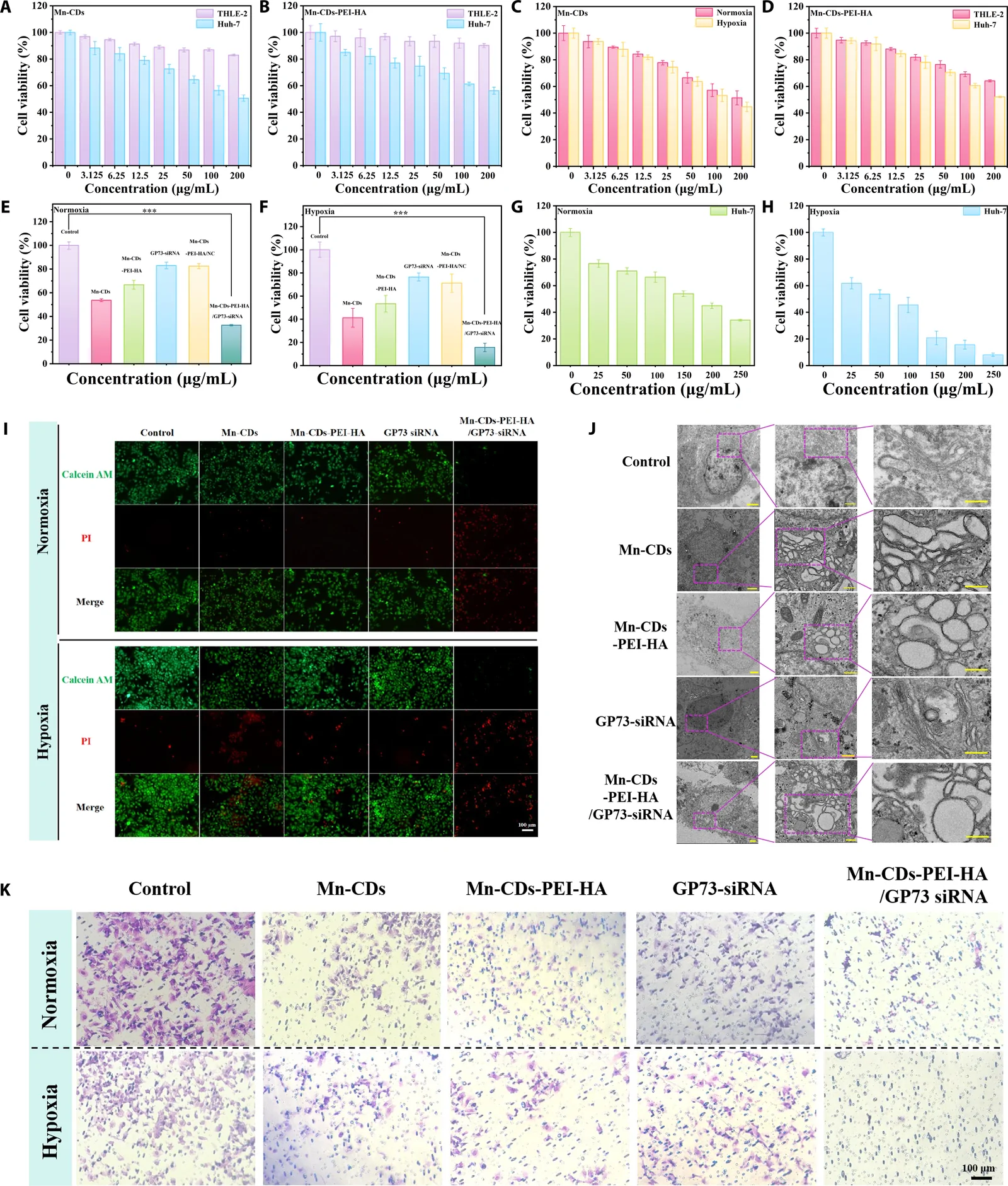
Cell viability of (A) Mn-CDs and (B) Mn-CDs-PEI-HA in normal THLE-2 cells and HCC Huh-7 cells; cell viability of Huh-7 cells under (C) normoxic and (D) hypoxic conditions; cell viability of Huh-7 cells treated with Mn-CDs, Mn-CDs-PEI-HA, and Mn-CDs-PEI-HA/GP73-siRNA (200 μg/mL) under (E) normoxic and (F) hypoxic conditions; cell viability of Huh-7 cells treated with different concentrations of Mn-CDs-PEI-HA/GP73-siRNA under (G) normoxic and (H) hypoxic conditions; (I) live/dead cell staining of Huh-7 cells after 24-h incubation with Mn-CDs, Mn-CDs-PEI-HA, and Mn-CDs-PEI-HA/GP73-siRNA under normoxic and hypoxic conditions; (J) bio-TEM images of the GA in Huh-7 cells after 24-h incubation with Mn-CDs, Mn-CDs-PEI-HA, and Mn-CDs-PEI-HA/GP73-siRNA; (K) microscopic images of Transwell invasion assays for Mn-CDs, Mn-CDs-PEI-HA, GP73-siRNA, and Mn-CDs-PEI-HA/GP73-siRNA under normoxic and hypoxic conditions. Data are presented as mean ± SD. ***P < 0.001. PI, propidium iodide.

To further validate the inhibitory effects of Mn-CDs, Mn-CDs-PEI-HA, and Mn-CDs-PEI-HA/GP73-siRNA on the proliferation of HCC cells in vitro, live/dead cell staining was employed to observe cell death under different treatments [46] (Fig. 7I). In the control and GP73-siRNA groups, all Huh-7 cells exhibited green FL, indicating minimal damage caused by high-glucose DMEM alone or GP73-siRNA alone, suggesting that GP73-siRNA lacks protection in the extracellular environment, is susceptible to degradation by enzymatic in vivo, and consequently fails to effectively enter cells to exert a therapeutic effect. The Mn-CDs and Mn-CDs-PEI-HA groups showed partial red FL, indicating that the carriers possess the ability to ameliorate hypoxia and exert a certain degree of tumor-killing effect. In contrast, the Mn-CDs-PEI-HA/GP73-siRNA group displayed the brightest red FL in the imaging, demonstrating a significant inhibitory effect on Huh-7 cell viability. As shown in Fig. S5A and B, under hypoxic conditions, the PI red FL intensity of Mn-CDs was markedly stronger than under normoxic conditions, indicating that Mn-CDs can, to some extent, ameliorate the tumor hypoxic microenvironment and thereby kill tumor cells. Compared with normoxic conditions, the Mn-CDs-PEI-HA/GP73-siRNA group exhibited significantly enhanced PI red FL intensity under hypoxia, confirming that Mn-CDs-PEI-HA/GP73-siRNA has excellent integrated tumor-killing efficacy, combining hypoxia amelioration with siRNA-mediated gene silencing.

To evaluate the siRNA-mediated gene-silencing efficacy of the carrier and complex, as well as their impact on the GA in Huh-7 cells, morphological changes of the GA were observed using bio-TEM [47] (Fig. 7J). The results show that the GA in the control group exhibited flattened and stacked morphology, while the GP73-siRNA-alone group induced only slight swelling and no significant alteration in GA morphology. In contrast, Mn-CDs, Mn-CDs-PEI-HA, and Mn-CDs-PEI-HA/GP73-siRNA groups induced substantial changes in the GA structure of Huh-7 cells, characterized by marked swelling and fragmentation of the GA. These findings indicate that Mn-CDs, Mn-CDs-PEI-HA, and Mn-CDs-PEI-HA/GP73-siRNA can target the GA and induce its disruption through hypoxia amelioration and gene-silencing therapy.

Since down-regulation of GP73 can inhibit tumor invasion and metastasis, the anti-invasive and anti-metastatic effects of Mn-CDs-PEI-HA/GP73-siRNA were evaluated using Transwell cell invasion assays and wound healing assays. The Transwell chamber primarily mimics the in vivo extracellular matrix environment to assess cell invasive capability. The Transwell system consists of upper and lower chambers. Typically, Huh-7 cells seeded in the upper chamber can migrate through the polyethylene terephthalate membrane of the Transwell into the lower chamber, which contains a complete medium with chemoattractants (serum). The number of cells that migrate to the lower chamber is then counted to reflect the invasive ability of the tumor cells [48]. As shown in Fig. 7K and Fig. S6A, numerous Huh-7 cells in the control group migrated through the permeable membrane, demonstrating high invasive ability. In contrast, the number of Huh-7 cells in the treatment groups was significantly reduced. Quantitative analysis revealed that under normoxic and hypoxic conditions, the invasion rates of the Mn-CDs-PEI-HA/GP73-siRNA group were 14% and 1%, respectively, which were significantly lower than those of the Mn-CDs and Mn-CDs-PEI-HA groups under normoxic conditions (47% and 53%) and hypoxic conditions (34% and 49%). These results indicate that Mn-CDs-PEI-HA/GP73-siRNA can synergistically inhibit the migration and invasion of Huh-7 cells by ameliorating hypoxia and suppressing GP73 expression.

Furthermore, as shown in Fig. S6B, after 24 h of culture, cells in the control group exhibited a significant tendency to migrate toward the center, indicating normal migratory capacity. Additionally, under both normoxic and hypoxic conditions, Mn-CDs, Mn-CDs-PEI-HA, and Mn-CDs-PEI-HA/GP73-siRNA group all demonstrated significant anti-migratory activity, indicating that both the carrier and complex possess anti-migratory capabilities.

In summary, the Mn-CDs-PEI-HA/GP73-siRNA exhibits excellent in vitro antitumor performance and greater inhibitory effect under hypoxic conditions than under normoxic conditions, demonstrating that they have dual therapeutic efficacy of ameliorating hypoxia and mediating gene silencing, and holds promising application prospects.

### In vivo antitumor activity of the complex

Given that the complex demonstrated excellent hypoxia-alleviating and gene-silencing therapeutic effects in vitro, the feasibility and effectiveness of the complex for in vivo tumor treatment were subsequently evaluated. As shown in Fig. 8A, a tumor-bearing mouse model was established by subcutaneously inoculating Hepa1-6 cells into the flank of mice, which were then divided into the following 5 groups: control group (G1), Mn-CDs group (G2), Mn-CDs-PEI-HA group (G3), GP73-siRNA group (G4), and Mn-CDs-PEI-HA/GP73-siRNA group (G5). Tumor-bearing mice were treated, and their body weight was recorded every 3 d. Results showed no significant changes in body weight in any group during the treatment period, and the mouse in the Mn-CDs-PEI-HA/GP73-siRNA group (G5) exhibited the highest body weight (Fig. 8B), indicating that the tumor treatment did not cause obvious side effects. Figure 8C shows the tumor volume change curves of each group during the treatment period. It can be observed that the tumor volume in the GP73-siRNA group (G4) was smaller than that in the control group (G1), indicating a certain siRNA-mediated gene-silencing effect. The tumor volume in the Mn-CDs group (G2) was smaller than that in the Mn-CDs-PEI-HA group (G3), which is attributed to the higher O_2_ production by Mn-CDs at the same concentration compared to Mn-CDs-PEI-HA (Fig. S1H), thereby ameliorating the hypoxic microenvironment of HCC and inhibiting HCC proliferation. Compared with the Mn-CDs group (G2) and the Mn-CDs-PEI-HA group (G3), the Mn-CDs-PEI-HA/GP73-siRNA group (G5) exhibited the smallest tumor volume. As observed from the sizes of the solid tumors in each group in Fig. 8D, compared with the control group (G1) and the GP73-siRNA group (G4), the Mn-CDs group (G2), Mn-CDs-PEI-HA group (G3), and Mn-CDs-PEI-HA/GP73-siRNA group (G5) demonstrated more pronounced therapeutic effects, with tumor inhibition rates of 54%, 41%, and 84%, respectively. The Mn-CDs group (G2) and Mn-CDs-PEI-HA group (G3) inhibited tumor growth solely by ameliorating hypoxia, whereas the Mn-CDs-PEI-HA/GP73-siRNA group (G5) synergistically suppressed HCC progression through the dual mechanisms of hypoxia amelioration and gene silencing, achieving significant tumor inhibitory outcome. After the treatment period, blood was collected from the mouse eyeballs for biochemical analysis. As shown in Fig. 8E, which displays the serum levels of white blood cell (WBC), lymph, RBC, and platelet (PLT) in the tumor-bearing mice, the indices for all groups fell within the normal range, indicating that neither the carrier nor the complex adversely affected the mice’s hematological parameters. In Fig. 8F, the values of ALT, AST, UREA, and CREA in the tumor-bearing mice were all within normal limits, demonstrating that none of the treatments induced hepatic or renal dysfunction. Furthermore, H&E staining results (Fig. 8G) revealed that during the treatment period, the major organs (heart, liver, spleen, lungs, and kidneys) of the mice exhibited tightly arranged cells with distinct nuclei and no significant damage, indicating that Mn-CDs, Mn-CDs-PEI-HA, and Mn-CDs-PEI-HA/GP73-siRNA all possess a high degree of biosafety and promising application prospects.

**Fig. 8.**
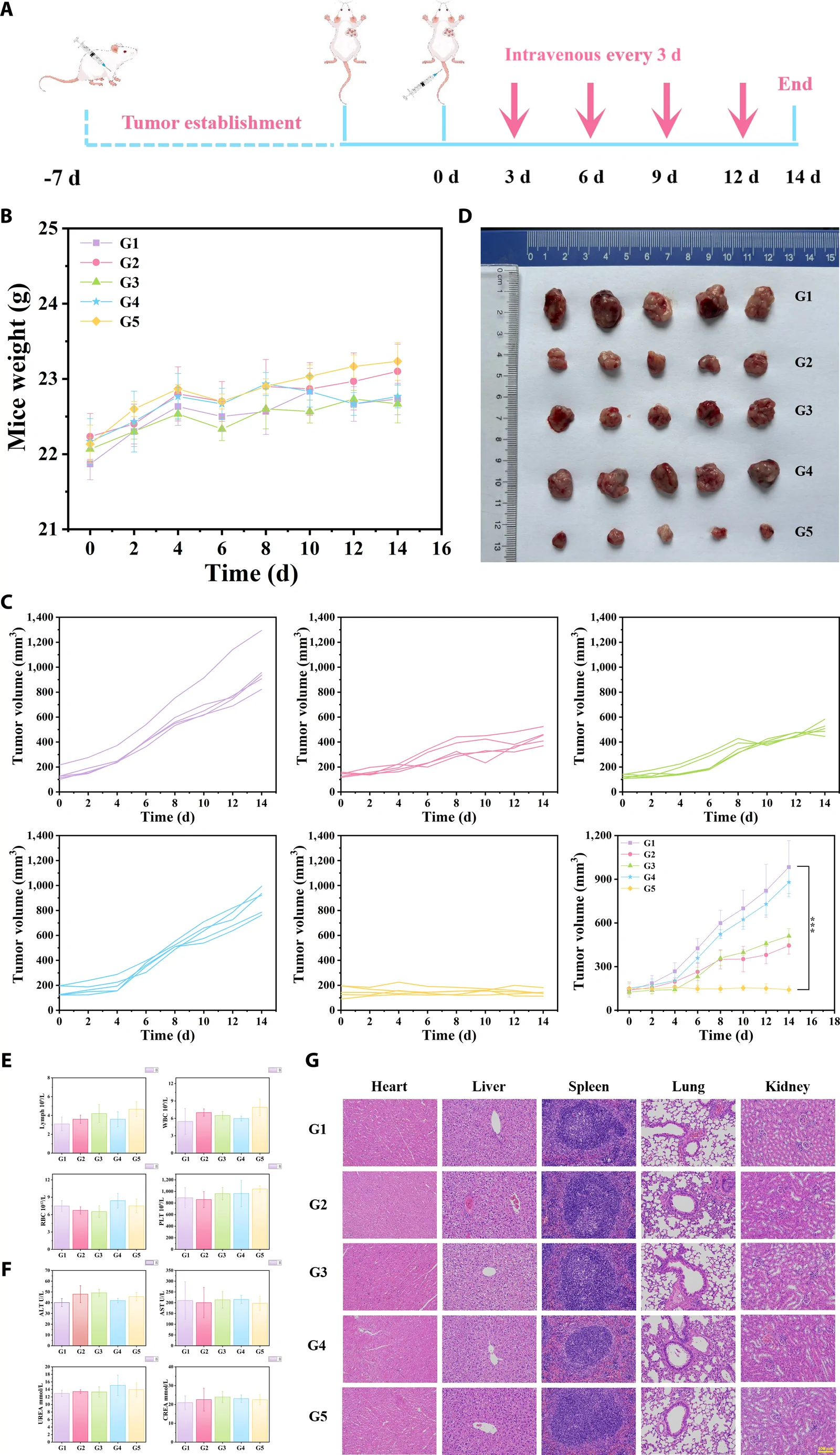
(A) Treatment schedule for tumor-bearing mice; (B) body weight curves and (C) tumor volume growth curves of each group during the treatment period; (D) photographs of excised solid tumors after treatment; (E) serum levels of lymph, WBC, RBC, and PLT, and (F) levels of ALT and AST (liver function) as well as UREA and CREA (renal function) in tumor-bearing mice after treatment; (G) H&E staining of major organs (heart, liver, spleen, lungs, and kidneys) from tumor-bearing mice after treatment. Data are presented as mean ± SD. ***P < 0.001.

In summary, as shown in Fig. 9, the HA and Cys residues on the surface of Mn-CDs-PEI-HA/GP73-siRNA confer dual-targeting capabilities for active targeting of HCC and GA, while the Mn^2+^ imparts CAT-like activity, enabling it to catalyze endogenous H_2_O_2_ in HCC to produce O_2_ and ameliorate the tumor hypoxic microenvironment. The incorporated GP73-siRNA down-regulates GP73 expression through siRNA-mediated gene silencing, allowing Mn-CDs-PEI-HA/GP73-siRNA to synergistically inhibit HCC via the dual therapeutic effects of hypoxia amelioration and gene silencing, thereby achieving favorable treatment outcomes. Furthermore, it can be utilized for FL/MR dual-modality imaging of HCC. Moreover, it is acknowledged that the current study does not include a systematic evaluation of GP73 expression under hypoxic conditions or its correlation with HIF-1α/CAIX/GLUT1/VEGF. Such investigations are essential for elucidating the hypoxia-driven regulatory network of GP73 and its impact on nanozyme targeting. Future work will focus on quantifying these markers under controlled oxygen conditions, which would further validate the hypoxia-dependent targeting mechanism and provide a more comprehensive understanding of the nanozyme’s behavior in the tumor microenvironment.

**Fig. 9.**
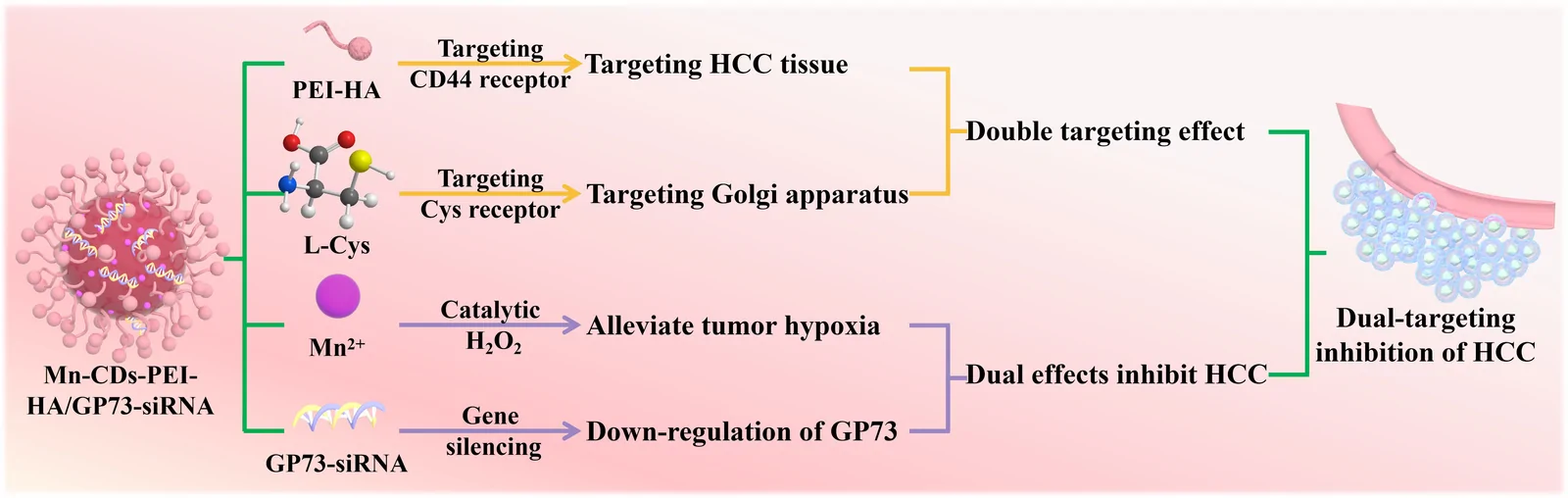
The mechanism diagram of the Mn-CDs-PEI-HA/GP73-siRNA.

## Conclusion

To sum up, based on the GP73–hypoxia positive feedback loop driving HCC progression, this study designed and synthesized a complex carrying genes, Mn-CDs-PEI-HA/GP73-siRNA. This complex can catalyze H_2_O_2_ to produce O_2_ under an acidic tumor microenvironment, exhibit CAT-like activity, and can actively target the GA of HCC cells for FL/MR dual-modality imaging. Both in vivo and in vitro experiments demonstrate that, compared with the GP73-siRNA (gene silencing) group and the Mn-CDs-PEI-HA (hypoxia alleviation) group, Mn-CDs-PEI-HA/GP73-siRNA achieves a high tumor inhibition rate of up to 84% in HCC treatment because Mn-CDs-PEI-HA/GP73-siRNA has excellent targeting ability and CAT-like activity, which enable precise delivery of GP73-siRNA to HCC cells, effectively protect GP73-siRNA from enzymatic degradation, and exert dual inhibitory effects through “hypoxia alleviation + gene silencing”. This multifunctional nanoplatform provides a novel strategy for the precise diagnosis and treatment of HCC.

## Data Availability

The authors confirm that the data supporting the findings of this study are available within the article.

## References

[1] Qu Y, Chen C, Sun F, Liu S, Zhao G, Zhao Z, Liu C, Jiang X, Li T. Epigenetic modulation with nanosatellite triggers tumoricidal immunity for hepatocellular carcinoma treatment. Nat Commun. 2025;16(1):7340. 10.1038/s41467-025-61974-w PMC12334573 40781069

[2] Cui L, Cui Y, Liu J, Li W, Wu M, Wei X, Lai Y, Mi P. Bioengineered nanovesicles for efficient siRNA delivery through ligand-receptor-mediated and enzyme-controlled membrane fusion. Nat Commun. 2025;16(1):6174. 10.1038/s41467-025-61230-1 PMC12227568 40615402

[3] Pu Y, Zhu C, Liao J, Gong L, Wu Y, Liu S, Wang H, Zhang Q, Lin Z. Antiviral nanomedicine: Advantages, mechanisms and advanced therapies. Bioact Mater. 2025;52:92–122. 10.1016/j.bioactmat.2025.05.030 PMC12173073 40530413

[4] Howley BV, Howe PH. Metastasis-associated upregulation of ER-Golgi trafficking kinetics: Regulation of cancer progression via the Golgi apparatus. Oncoscience. 2018;5(5–6):142–143. 10.18632/oncoscience.426 PMC6049300 30035168

[5] Liu Y, Hu X, Liu S, Zhou S, Chen Z, Jin H. Golgi phosphoprotein 73: The driver of epithelial-mesenchymal transition in cancer. Front Oncol. 2021;11: Article 783860. 10.3389/fonc.2021.783860 PMC8688837 34950590

[6] Liu Y, Hu X, Zhou S, Sun T, Shen F, Zeng L. Golgi protein 73 promotes angiogenesis in hepatocellular carcinoma. Research. 2024;7:0425. 10.34133/research.0425 PMC11251733 39022745

[7] Bajaj R, Warner AN, Fradette JF, Gibbons DL. Dance of the Golgi: Understanding Golgi dynamics in cancer metastasis. Cells. 2022;11:1484. 10.3390/cells11091484 PMC9102947 35563790

[8] Zhang Z, Yan L, Li W, Hu J, Wang Y, Dou H, Pan D, Shen L, Shi S, Geng B. D-π-A sensitized carbon dots as long-lived type-I/II photosensitizers for NIR-excited hypoxia-regulated photodynamic therapy. Nat Commun. 2026;17:4756. 10.1038/s41467-026-71476-y PMC13216253 41927538

[9] Xiao L, Cai J, Wang Y, Hu J, Dou H, Pan D, Geng B, Cheng P, Shen L. Zr-based metal-organic framework dual-sensitized by carbon dot sonosensitizers and MnO_2-x_ nanozymes for cGAS-STING activation enhanced sonocatalytic-immunotherapy. Adv Funct Mater. 2026;36(25): Article e19998.

[10] Hu J, Xiang Y, Zhang Z, Cai J, Wang Y, Dou H, Pan D, Shen L, Geng B. Atomic-valence engineering of a Mn(III)-tuned sonozyme system for multimodal tumor immunotherapy. Biomaterials. 2026;329: Article 123987. 10.1016/j.biomaterials.2026.123987 41512497

[11] Zhao J, Cai J, Hu J, Zhang Z, Liu Y-Y, Pan D, Shen L, Geng B. Biodegradable hollow MnO_2_ decorated by carbon dots with cholesterol depletion capability for cascaded amplification of sono-immunotherapy. Biomaterials. 2026;325: Article 123559. 10.1016/j.biomaterials.2025.123559 40680707

[12] Wang F, Dong X, Zuo Y, Xie Z, Guan R. Effectively enhancing red fluorescence strategy and bioimaging applications of carbon dots. Mater Today Phys. 2024;41: Article 101332.

[13] Xu H, Chang J, Wu H, Wang H, Xie W, Li Y, Li X, Zhang Y, Fan L. Carbon dots with guanidinium and amino acid functional groups for targeted small interfering RNA delivery toward tumor gene therapy. Small. 2023;19(31): Article e2207204. 10.1002/smll.202207204 36840641

[14] Xue W, Du J, Wei Y, Zhou S, Wang S, Yang Y, Chen L, Yu S. Construction of cationic carbon dots carrying survivin siRNA delivery system and its active targeting therapy for liver cancer. Int J Nanomedicine. 2025;20:6831–6851. 10.2147/IJN.S521972 PMC12126988 40452787

[15] Wei YY, Chen L, Zhang X, Du JL, Li Q, Luo J, Liu XG, Yang YZ, Yu SP, Gao YD. Orange-emissive carbon quantum dots for ligand-directed Golgi apparatus-targeting and *in vivo* imaging. Biomater Sci. 2022;10(15):4345–4355. 10.1039/d2bm00429a 35781543

[16] Li R, Wei F, Wu X, Zhou P, Chen Q, Cen Y, Xu G, Cheng X, Zhang A, Hu Q. PEI modified orange emissive carbon dots with excitation-independent fluorescence emission for cellular imaging and siRNA delivery. Carbon. 2021;177:403–411.

[17] Chiesa E, Greco A, Riva F, Dorati R, Conti B, Modena T, Genta I. CD44-targeted carriers: The role of molecular weight of hyaluronic acid in the uptake of hyaluronic acid-based nanoparticles. Pharmaceuticals. 2022;15(1):103. 10.3390/ph15010103 PMC8781203 35056160

[18] Wang M, Zhu H, Liu B, Hu P, Pan J, Niu X. Bifunctional Mn-doped N-rich carbon dots with tunable photoluminescence and oxidase-mimetic activity enabling bimodal ratiometric colorimetric/fluorometric detection of nitrite. ACS Appl Mater Interfaces. 2022;14(39):44762–44771. 10.1021/acsami.2c14045 36129748

[19] Sun S, Zhao L, Wu D, Zhang H, Lian H, Zhao X, Wu A, Zeng L. Manganese-doped carbon dots with redshifted orange emission for enhanced fluorescence and magnetic resonance imaging. ACS Appl Bio Mater. 2021;4(2):1969–1975. 10.1021/acsabm.0c01597 35014466

[20] Jin P, Wang D, Lu Y, Qian Y, Fang C, Zhang X, Qian J, Qu S, Wang H. Low-coordination configuration single-atom manganese nanozymes for NIR-imaging-oriented efficient catalytic oncotherapy. Adv Sci. 2025;12(18): Article 2502664. 10.1002/advs.202502664 PMC12079523 40091543

[21] Peng J, Yu Z, Yang Y, Xie G, Wu S, Lin H, Lin Y. Regulating spatial distribution of silicon and graphite for high-performance anode materials via plasma-assisted ball milling. J Electroceram. 2025;53(2):257–267.

[22] Wang B, Wang H, Zhang B, Hu Y, Lu S. Hot exciton carbon dots-based deep blue electroluminescent light-emitting diodes exceeding 7% external quantum efficiency. Adv Funct Mater. 2024;34(44): Article 2404437.

[23] Geng B, Hu J, Li Y, Feng S, Pan D, Feng L, Shen L. Near-infrared phosphorescent carbon dots for sonodynamic precision tumor therapy. Nat Commun. 2022;13(1):5735. 10.1038/s41467-022-33474-8 PMC9523047 36175446

[24] Fu C, Duan X, Cao M, Jiang S, Ban X, Guo N, Zhang F, Mao J, Huyan T, Shen J, et al. Targeted magnetic resonance imaging and modulation of hypoxia with multifunctional hyaluronic acid-MnO_2_ nanoparticles in glioma. Adv Healthc Mater. 2019;8(10): Article 1900047. 10.1002/adhm.201900047 30920772

[25] Zhu W, Wang H, Feng B, Liu G, Bian Y, Zhao T, Wang Q, Weng X. Self-healing hyaluronic acid-based hydrogel with miRNA140-5p loaded MON-PEI nanoparticles for chondrocyte regeneration: Schiff base self-assembly approach. Adv Sci. 2025;12(1): Article 2406479. 10.1002/advs.202406479 PMC11714154 39498998

[26] Xie W, Hao Q, Ye Z, Sha R, Wen B, Wang H, Zhang H, Jia G, Le XC, Jiang G, et al. Spherical nucleic acids-directed cryosynthesis of manganese nanoagents for tumor imaging and therapy. Angew Chem Int Ed Engl. 2025;64(24): Article e202503004. 10.1002/anie.202503004 40178305

[27] Wang L, Wang B, Liu E, Zhao Y, He B, Wang C, Xing G, Tang Z, Zhou Y, Qu S. Polyetherimide functionalized carbon dots with enhanced red emission in aqueous solution for bioimaging. Chin Chem Lett. 2022;33(8):4111–4115.

[28] Deng X, Wu S, Zang S, Liu X, Ma Y. PDA–PEI-copolymerized nanodots with tailorable fluorescence emission and quenching properties for the sensitive ratiometric fluorescence sensing of miRNA in serum. Anal Chem. 2022;94(42):14546–14553. 10.1021/acs.analchem.2c02156 36215706

[29] Peng Y, Liang S, Liu D, Ma K, Yun K, Zhou M, Hai L, Xu M, Chen Y, Wang Z. Multi-metallic nanosheets reshaping immunosuppressive tumor microenvironment through augmenting cGAS-STING innate activation and adaptive immune responses for cancer immunotherapy. Adv Sci. 2024;11(38): Article e2403347. 10.1002/advs.202403347 PMC11481177 39120546

[30] Gan J, Lei J, Li Y, Lu M, Yu X, Yu G. Manganese oxide-incorporated hybrid lipid nanoparticles amplify the potency of mRNA vaccine via oxygen generation and STING activation. J Am Chem Soc. 2024;146(47):32689–32700. 10.1021/jacs.4c12166 39552027

[31] Jia Q, Ge J, Liu W, Zheng X, Chen S, Wen Y, Zhang H, Wang P. A magnetofluorescent carbon dot assembly as an acidic H_2_O_2_ -driven oxygenerator to regulate tumor hypoxia for simultaneous bimodal imaging and enhanced photodynamic therapy. Adv Mater. 2018;30(13): Article e1706090. 10.1002/adma.201706090 29436031

[32] Liu J, Zhu Y, Fan Y, Gong L, Zhu X, Zhang Y, Liu M, Yao S. The pH-dependent multiple nanozyme activities of copper-cerium dioxide and its application in regulating intracellular oxygen and hydrogen peroxide levels. J Colloid Interface Sci. 2024;654(Pt B):1054–1062. 10.1016/j.jcis.2023.10.050 39491063

[33] Chen M, He H, Dong J, Xie P, Chen J, Ma L, Chen T. In situ oxygen generation by a low-toxicity ruthenium electrocatalyst for multimodal radiotherapy sensitization. Inorg Chem Front. 2025;12(5):1857–1866.

[34] Wang Q, Meng J, Huang L, Wu F, Yi X, Su G, Li Y, Hou Z, Fan Z. Platinum-coordinated engineered nanoreactors with O_2_ self-amplification and on-demand cascade chemo-drug synthesis for self-reinforcing hypoxic oncotherapy. ACS Appl Mater Interfaces. 2023;15(14):17495–17506. 10.1021/acsami.2c22153 36996342

[35] Zhu Y, Wang D, Du C, Wu T, Wei P, Zheng H, Li G, Zheng S, Su L, Yan L, et al. Ruthenium single-atom nanozyme driven sonosensitizer with oxygen vacancies enhances electron–hole separation efficacy and remodels tumor microenvironment for sonodynamic-amplified ferroptosis. Adv Sci. 2025;12(22): Article e2416997. 10.1002/advs.202416997 PMC12165091 40279631

[36] Phuong PT, Oliver S, He J, Wong EHH, Mathers RT, Boyer C. Effect of hydrophobic groups on antimicrobial and hemolytic activity: Developing a predictive tool for ternary antimicrobial polymers. Biomacromolecules. 2020;21(12):5241–5255. 10.1021/acs.biomac.0c01320 33186496

[37] Tao Y, Luo P, Jing F, Liu T, Tan X, Lyu Z, VeerleBernaerts K, Zhang T, Jia R. Collagen-inspired 3D printing electrospinning biomimetic patch for abdominal wall defect regeneration. Adv Fiber Mater. 2025;7(4):1177–1194.

[38] Zhou Y, Wu Y, Sun S, Wang W, Zhou S, Liu H, Guo Y, Hong S, Ding F, Cai H. Self-assembled glycopeptide as a biocompatible mRNA vaccine platform elicits robust antitumor immunity. ACS Nano. 2025;19(15):14727–14741. 10.1021/acsnano.4c15187 40203215

[39] Wang Z, Wei J, Liu Z, Huang X, In LP, He K, Ding Y-F, Wang R. Glutathione-depleting supramolecular gold nano-assemblies for NIR-II imaging guided photodynamic immunotherapy. Chem Eng J. 2025;507: Article 160427.

[40] Xi Y, Bai S, Tian Y, Lv Y, Ji L, Li W, He G, Yang L. Golgi-targeted NIR fluorescent probe with large Stokes shift for real-time monitoring of nitric oxide in depression model. Bioorg Chem. 2024;148: Article 107476. 10.1016/j.bioorg.2024.107476 38788368

[41] Li J, Tang J, Yang X, Xie P, Liu J, Zhang D, Ye Y. A novel aggregation-induced emission fluorescent probe to visualize peroxynitrite levels during Golgi stress. Sens Actuators B Chem. 2022;358: Article 131513.

[42] Li W, Xin H, Zhang Y, Feng C, Li Q, Kong D, Sun Z, Xu Z, Xiao J, Tian G, et al. NIR-II fluorescence imaging-guided oxygen self-sufficient nano-platform for precise enhanced photodynamic therapy. Small. 2022;18(51): Article e2205647. 10.1002/smll.202205647 36328734

[43] Gao F, Fu Q, Ruan Y, Li C, Wang Y, Li H, Li J, Jiang Y. Elucidating manganese single-atom doping: Strategies for fluorescence enhancement in water-soluble red-emitting carbon dots and applications for FL/MR dual mode imaging. Adv Sci. 2025;12(8): Article e2414895. 10.1002/advs.202414895 PMC11848584 39746856

[44] Lin T, Zhao X, Zhao S, Yu H, Cao W, Chen W, Wei H, Guo H. O_2_-generating MnO_2_ nanoparticles for enhanced photodynamic therapy of bladder cancer by ameliorating hypoxia. Theranostics. 2018;8(4):990–1004. 10.7150/thno.22465 PMC5817106 29463995

[45] Zhang X, Xu D-Z, Zhao W-J, Han X-Y, Lu Z-L, Liu R. A multifunctional polyester nanoplatform for the synergistic anticancer: Enhanced photodynamic therapy and targeted gene silencing. Angew Chem Int Ed Engl. 2025;64(33): Article e202505041. 10.1002/anie.202505041 40501173

[46] Hu Z-C, Wang B, Zhou X-G, Liang H-F, Liang B, Lu H-W, Ge Y-X, Chen Q, Tian Q-W, Xue F-F, et al. Golgi apparatus-targeted photodynamic therapy for enhancing tumor immunogenicity by eliciting NLRP3 protein-dependent pyroptosis. ACS Nano. 2023;17(21):21153–21169. 10.1021/acsnano.3c05005 37921421

[47] Shi M, Fu Z, Pan W, Wang K, Liu X, Li N, Tang B. A Golgi apparatus-targeted photothermal agent with protein anchoring for enhanced cancer photothermal therapy. Adv Healthc Mater. 2024;13(17): Article e2303749. 10.1002/adhm.202303749 38483042

[48] He J, Wang Y, Ren Y, Yuan Q, Zhang Z, Li L, Bao B, Jia W, Zhang X, Li M, et al. Calcium-mediated cell adhesion enhancement-based antimetastasis and synergistic antitumor therapy by conjugated polymer–calcium composite nanoparticles. ACS Nano. 2024;18(36):24953–24967. 10.1021/acsnano.4c05771 39197151

