# Supplementary material for: Application of Dual-Targeting Carbon Dot Nanozymes in Improving Hypoxia and Enhancing Small Interfering RNA-Mediated Gene Silencing for Hepatocellular Carcinoma
Source: Biomater Res. 2026 Oct 19;30:0430. doi: 10.34133/bmr.0430 (PMC13678084; doi:10.34133/bmr.0430)
Supplement: Supplementary file 1 [file bmr.0430.f1.docx]

Application of dual-targeting carbon dot nanozymes in improving hypoxia and enhancing small interfering RNA-mediated gene silencing

for hepatocellular carcinoma

Yingying Wei, ^#1,2^ Yangyi Cao, ^#3^ Wencheng Shao, ^4^ Tao Zhang, ^5^ Xiaobo Zhang, ^6^ Wenhui Dong, ^4^ Shizhao Zhou, ^4^ Yongzhen Yang, ^*7, 8^ Lin Chen, ^*7^ Shiping Yu, ^*1^

1. Department of Interventional Therapy, Shanxi Province Cancer Hospital, Shanxi Hospital Affiliated to Cancer Hospital, Chinese Academy of Medical Sciences, Cancer Hospital Affiliated to Shanxi Medical University, Taiyuan 030000, P. R. China.
2. Cancer Hospital Affiliated, Shanxi Medical University, Taiyuan 030000, P. R. China
3. The Department of Radiology, Renmin Hospital of Wuhan University, Wuhan 430060, P. R. China
4. Medical Imaging Department, Shanxi Medical University, Taiyuan 030001, P. R. China
5. Shanxi Academy of Medical Sciences, Shanxi Medical University, Taiyuan 030001, P. R. China.
6. Department of Radiology, the First Medical Center of PLA General Hospital, Beijing P. R. 100039, China.
7. Key Laboratory of Interface Science and Engineering in Advanced Materials, Ministry of Education, Taiyuan University of Technology, Taiyuan 030024, P. R. China.
8. College of Textile Engineering, Taiyuan University of Technology, Taiyuan, 030024, P. R. China.

*Corresponding authors. E-mails: [yangyongzhen@tyut.edu.cn](mailto:yangyongzhen@tyut.edu.cn);

chenlin01@tyut.edu.cn; [yushiping@sxmu.edu.cn](mailto:yushiping@sxmu.edu.cn);

#These authors contributed equally to this work.

Contents

Fig. S1 (a) Mn 3s XPS spectrum of Mn-CDs; (b) Mn 2p and (c) Mn 3s XPS spectrum of Mn-CDs-PEI-HA; (d) the absorbance values of Mn-CDs at 536 nm in different media over different days; (e) FL intensity comparison after co-incubation of RDPP FL probe with Mn-CDs and Mn-CDs-PEI-HA; (f) schematic diagram of the O_2_ generation mechanisms for Mn-CDs and Mn-CDs-PEI-HA; (g) FL SNR analysis of control, Mn-CDs and Mn-CDs-PEI-HA groups; (h) dissolved O_2_ release curves of Mn-CDs and Mn-CDs-PEI-HA at the same concentration (200 μg/mL).

Fig. S2 *In vivo* FL imags of nude mice at different time points after intravenous injection of (a) Mn-CDs and (b) Mn-CDs-PEI-HA, ex *vivo* FL imaging of major organs, and the corresponding ex *vivo* FL intensity of liver and kidneys.

Fig. S3 (a) *Ex* *vivo* FLimages of the tumor, heart, liver, spleen, lung, and kidney from tumor-bearing mice at various times post-injection; (b) ex *vivo* FL intensity of the tumor.

Fig. S4 Effects of three GP73-siRNAs on GP73-mRNA expression.

Fig. S5 FL intensity corresponding to live/dead cell staining of Huh-7 cells after 24 h co-incubation with Mn-CDs, Mn-CDs-PEI-HA, and Mn-CDs-PEI-HA/GP73-siRNA under (a) normoxic and (b) hypoxic conditions.

Fig. S6 (a) Transwell invasion quantitative histogram and (b) scratch wound healing microscopy images of Mn-CDs, Mn-CDs-PEI-HA, GP73-siRNA, and Mn-CDs-PEI-HA/GP73-siRNA under normoxic and hypoxic conditions.

Stability of Mn-CD nanozyme

To evaluate the stability of Mn-CD nanozyme, the Mn-CD nanozyme was dissolved in normal saline, neutral PBS, acidic PBS, basal medium, and complete medium, and their absorbance at 536 nm was measured on days 1, 3, and 7.

**a**

**b**

**c**

**d**

**e**

**f**

**g**

**h**

Fig. S1 (a) Mn 3s XPS spectrum of Mn-CDs; (b) Mn 2p and (c) Mn 3s XPS spectrum of Mn-CDs-PEI-HA; (d) the absorbance values of Mn-CDs at 536 nm in different media over different days; (e) FL intensity comparison after co-incubation of RDPP FL probe with Mn-CDs and Mn-CDs-PEI-HA; (f) schematic diagram of the O_2_ generation mechanisms for Mn-CDs and Mn-CDs-PEI-HA; (g) FL SNR analysis of control, Mn-CDs and Mn-CDs-PEI-HA groups; (h) dissolved O_2_ release curves of Mn-CDs and Mn-CDs-PEI-HA at the same concentration (200 μg/mL).

**a**

**b**

Fig. S2 *In vivo* FL images of nude mice at different time points after intravenous injection of (a) Mn-CDs and (b) Mn-CDs-PEI-HA, ex *vivo* FL imaging of major organs, and the corresponding ex *vivo* FL intensity of liver and kidneys.

**a**

**b**

Fig. S3 (a) *Ex* *vivo* FL images of the tumor, heart, liver, spleen, lung, and kidney from tumor-bearing mice at various times post-injection of Mn-CDs-PEI-HA; (b) ex *vivo* FL intensity of the tumor.

Fig. S4 Effects of three GP73-siRNAs on GP73-mRNA expression.

**a**

**b**

Fig. S5 FL intensity corresponding to live/dead cell staining of Huh-7 cells after 24 h co-incubation with Mn-CDs, Mn-CDs-PEI-HA, and Mn-CDs-PEI-HA/GP73-siRNA under (a) normoxic and (b) hypoxic conditions.

**a**

**b**

Fig S6 (a) Transwell invasion quantitative histogram and (b) scratch wound healing microscopy images of Mn-CDs, Mn-CDs-PEI-HA, GP73-siRNA, and Mn-CDs-PEI-HA/GP73-siRNA under normoxic and hypoxic conditions.
